# Dynamic MAPK signaling activity underlies a transition from growth arrest to proliferation in *Drosophila scribble* mutant tumors

**DOI:** 10.1242/dmm.040147

**Published:** 2019-08-29

**Authors:** Tiantian Ji, Lina Zhang, Mingxi Deng, Shengshuo Huang, Ying Wang, Tri Thanh Pham, Andrew Alan Smith, Varun Sridhar, Clemens Cabernard, Jiguang Wang, Yan Yan

**Affiliations:** 1 Division of Life Science, Hong Kong University of Science and Technology, Clear Water Bay, Kowloon, Hong Kong, China; 2 Center of Systems Biology and Human Health, School of Science and Institute for Advanced Study, Hong Kong University of Science and Technology, Clear Water Bay, Kowloon, Hong Kong, China; 3 Department of Chemical and Biological Engineering, Hong Kong University of Science and Technology, Clear Water Bay, Kowloon, Hong Kong, China; 4 Department of Biology, University of Washington, Life Science Building, Seattle, WA 98195, USA

**Keywords:** Cell polarity, *Drosophila* tumor model, JNK, ERK

## Abstract

Human tumors exhibit plasticity and evolving capacity over time. It is difficult to study the mechanisms of how tumors change over time in human patients, in particular during the early stages when a few oncogenic cells are barely detectable. Here, we used a *Drosophila* tumor model caused by loss of *scribble* (*scrib*), a highly conserved apicobasal cell polarity gene, to investigate the spatial-temporal dynamics of early tumorigenesis events. The fly *scrib* mutant tumors have been successfully used to model many aspects of tumorigenesis processes. However, it is still unknown whether *Drosophila scrib* mutant tumors exhibit plasticity and evolvability along the temporal axis. We found that *scrib* mutant tumors displayed different growth rates and cell cycle profiles over time, indicative of a growth arrest-to-proliferation transition as the *scrib* mutant tumors progress. Longitudinal bulk and single-cell transcriptomic analysis of *scrib* mutant tumors revealed that the MAPK pathway, including JNK and ERK signaling activities, showed quantitative changes over time. We found that high JNK signaling activity caused G2/M cell cycle arrest in early *scrib* mutant tumors. In addition, JNK signaling activity displayed a radial polarity with the JNK^high^ cells located at the periphery of *scrib* mutant tumors, providing an inherent mechanism that leads to an overall decrease in JNK signaling activity over time. We also found that ERK signaling activity, in contrast to JNK activity, increased over time and promoted growth in late-stage *scrib* mutant tumors. Furthermore, high JNK signaling activity repressed ERK signaling activity in early *scrib* mutant tumors. Together, these data demonstrate that dynamic MAPK signaling activity, fueled by intratumor heterogeneity derived from tissue topological differences, drives a growth arrest-to-proliferation transition in *scrib* mutant tumors.

This article has an associated First Person interview with the joint first authors of the paper.

## Abstract (precis)

**Summary:** The authors provide evidence to show that a well-established *Drosophila* tumor model, caused by loss of apicobasal cell polarity, harbors a surprising degree of plasticity and evolvability along the temporal axis.

## INTRODUCTION

Proteins essential for maintaining epithelial structures, such as cell polarity complexes, are involved in growth control (Bilder, 2004; Boggiano and Fehon, 2012; Sun and Irvine, 2016). For example, the basolateral Scribble complex, composed of Scribble (Scrib), Discs large (Dlg) and Lethal giant larvae [L(2)gl], was discovered as a group of ‘neoplastic tumor suppressor genes’ (nTSGs) in *Drosophila* (Bilder et al., 2000; Gateff, 1978; Woods and Bryant, 1991). *Drosophila* larvae homozygous mutants for any of the nTSGs grow into giant larvae with tumorous imaginal discs and optic lobes. These mutant tumors fail to differentiate and grow into masses that survive serial transplantations, induce cachexia and eventually kill the hosts (Figueroa-Clarevega and Bilder, 2015; Gateff, 1978). Studies of *Drosophila* nTSGs over decades have provided valuable insights into the mechanisms of growth control and tumorigenesis (Bilder, 2004; Gonzalez, 2013; Pastor-Pareja and Xu, 2013; Richardson and Portela, 2018; Sonoshita and Cagan, 2017). For example, analyses of nTSG mutant clonal growth have revealed the cooperative actions of multiple conserved signaling pathways during tumor development, cell competition-mediated tumor suppression mechanisms and tumor microenvironment influences (Brumby and Richardson, 2003; Chen et al., 2012; Cordero et al., 2010; Igaki et al., 2006; Katheder et al., 2017; Pagliarini and Xu, 2003; Vaughen and Igaki, 2016; Yamamoto et al., 2017). In particular, JNK and ERK signaling pathways, subgroups of the MAPK pathway (frequently deregulated in human cancers), have long been known as crucial for switching the growth outcome of *scrib* mutant cells when they are generated as mosaic loss-of-function clones surrounded by wild-type cells (Brumby and Richardson, 2003; Cordero et al., 2010; Igaki et al., 2006; Pagliarini and Xu, 2003; Uhlirova and Bohmann, 2006).

Interestingly, although *Drosophila* nTSG mutant tumors have successfully modeled many aspects of human epithelial cancers, it remains unclear whether and how the fast-growing fly nTSG mutant tumors change over time. The majority of mammalian tumors undergo evolution fueled by intratumor heterogeneity (McGranahan and Swanton, 2017). Many sources can contribute to intratumor heterogeneity. For example, individual tumor cells can acquire different types of mutations and grow into distinct subclones within the same tumor (de Bruin et al., 2014; Gerlinger et al., 2012). Moreover, most tumor cells co-exist with various immune and stromal cells, and different niches further contribute to intratumor heterogeneity (Jimenez-Sanchez et al., 2017). Whether the fly *scrib* mutant tumors exhibit some degree of intratumor heterogeneity is unclear.

Through quantitative analysis of *Drosophila scrib* mutant tumor growth, we found that over time *scrib* mutant tumors display different growth rates and cell cycle profiles. Moreover, quantitative changes in MAPK signaling activity underlie the change in growth rate. In particular, high JNK signaling activity is a primary cause of growth arrest in early *scrib* mutant tumors. We further found that JNK signaling activation is highly heterogeneous in *scrib* mutant tumors. JNK signaling activity displays a radial polarity, with JNK^high^ cells located at the tumor periphery, which underlies a decrease in JNK signaling activity over time. Together, these data reveal that fly *scrib* mutant tumors are highly dynamic on the temporal axis.

## RESULTS

### The *scrib* mutant tumors display different growth rates and cell cycle profiles over time

**Fig. 1.**
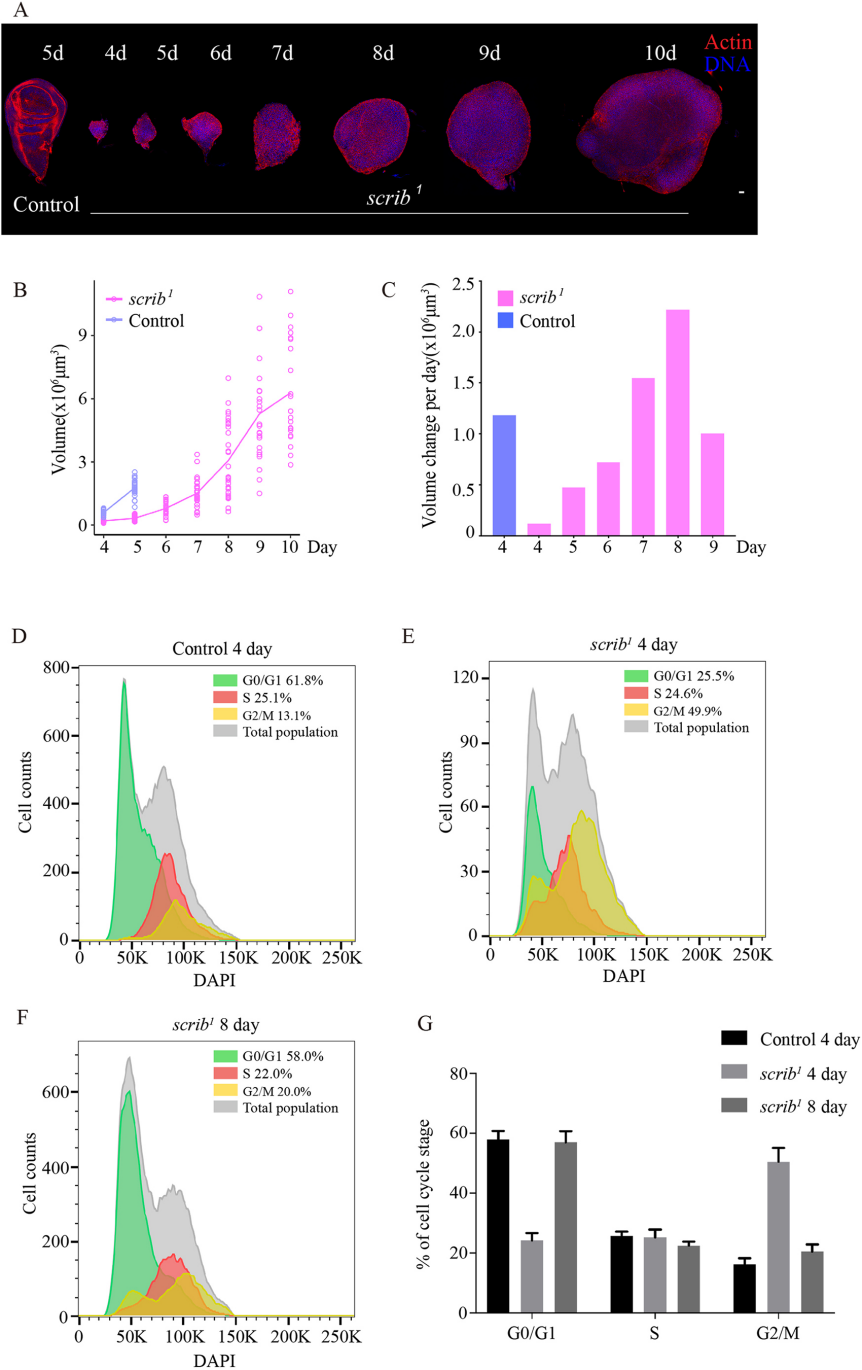
**The *scrib* mutant cells display different growth rates and cell cycle profiles over time.** (A) Examples of a control imaginal disc collected at 5 days AEL and *scrib^1^* mutant wing imaginal discs at 4-10 days AEL, stained for actin (red) and DNA (blue). Control genotype was *FRT82B*. Scale bar: 20 μm. (B) Quantification of volumes for control and *scrib^1^* mutant wing imaginal discs over time. Control genotype was *FRT82B* raised under identical conditions. Control, 4 days *n*=20, 6±1×10^5^ μm^3^, 5 days *n*=27, 1.8±0.4×10^6^ μm^3^; *scrib^1^* mutant, 4 days *n*=22, 2±1×10^5^ μm^3^, 5 days *n*=30, 3±1×10^5^ μm^3^, 6 days *n*=26, 8±3×10^5^ μm^3^, 7 days *n*=29, 1.5±0.7×10^6^ μm^3^, 8 days *n*=31, 3±2×10^6^ μm^3^, 9 days *n*=24, 5±2×10^6^ μm^3^, 10 days *n*=22, 6±2×10^6^ μm^3^. Note that larvae from the control group become pupae at 5 days AEL. (C) Quantification of volume change per day for control and *scrib^1^* mutant wing imaginal discs over time. Control genotype was *FRT82B* raised under identical conditions. (D-F) FACS analysis of cell cycle profiles of controls at 4 days AEL (D) and *scrib^1^* mutant wing imaginal disc cells at 4 (E) and 8 (F) days AEL. Genotype of the control group was *Ubi-GFP.E2f1 Ubi-mRFP1.CycB; FRT82B*. Genotype of the *scrib* mutant group was *Ubi-GFP.E2f1 Ubi-mRFP1.CycB; scrib*^1^. At least 5000 cells were recorded for each cell group. Each experiment was replicated at least three times. (G) Average percentage of cells in G0/G1, S and G2/M phases. Error bars indicate standard deviation.

To explore temporal changes in *scrib* mutant tumors, we monitored the growth of wing imaginal discs derived from eggs collected from a *scrib^1^* allele over 3 h (Bilder et al., 2000) (Fig. 1A,B). We quantitatively measured the volumes of the *scrib* mutant tumors, which are known to grow over an extended larval period (Bunker et al., 2015; Vaccari and Bilder, 2005). On average, at 4 and 5 days after egg laying (AEL), the volumes of the *scrib* mutant tumors were around 15-30% of those of control imaginal discs raised under identical conditions (Fig. 1A,B). We calculated the growth rate (defined as the average volume change per day) in *scrib* mutant and wild-type imaginal discs based on the volume measurements in Fig. 1B and found that the growth rate of *scrib* mutant tumors increased over time and, starting from day 7, became comparable with that of the control group at 4 days AEL (Fig. 1C). Note that the *scrib* mutant tumors monitored in our experiments eventually grew to sizes consistent with previous reports (Bilder et al., 2000; Gateff, 1978).

To test whether the growth rate change we observed was specific to the *scrib^1^* allele, we also quantified the growth rate of tumors depleted of Scrib or Dlg protein at the posterior region of wing discs using *engrailed*-Gal4-mediated RNAi. At 4 and 5 days AEL, the volume of *scrib* RNAi and *dlg* RNAi tumors was much smaller than the size of the posterior region in control wing discs (Fig. S1). However, the anterior regions of the *scrib* RNAi, *dlg* RNAi and control imaginal discs had comparable volumes (Fig. S1), indicating that the growth arrest phenotype observed in early *scrib* tumors is likely to be inherent and independent of an overall delay in larval development. Interestingly, from 4 to 6 days AEL we detected an increase in phosphorylated histone H3-positive (PH3^+^) cell number in the posterior *scrib* RNAi and *dlg* RNAi tumors (Fig. S1), indicative of changing growth rates during tumor progression. The fly larvae harboring *scrib* RNAi or *dlg* RNAi imaginal discs turned into pupae by day 7 AEL, preventing further measurement of growth rates. Notably, the cell competition process mediated by Minute mutations and differential Myc levels does not cross compartment boundaries (Johnston, 2009; Morata and Ripoll, 1975; Simpson, 1979; Simpson and Morata, 1981). However, tumorigenic dCsk-deficient cells generated in the *patched* expression domain are known to undergo apoptosis at the anterior–posterior boundary (Vidal et al., 2006). It is therefore likely that the growth of posterior *scrib RNAi* and *dlg RNAi* tumors is largely independent of the influence of cell competition, except for cells at the anterior–posterior boundary.

It is possible to attribute the growth arrest phenotype of early *scrib* mutant tumors to increased apoptosis, defects in cellular growth or defective cell proliferation. However, we found that prevention of apoptosis by overexpressing p35 did not rescue the growth arrest of posterior *scrib* RNAi cells (Fig. S2). Moreover, although we were able to detect apoptotic cells in the posterior *scrib* RNAi region, a similar number of apoptotic cells could also be detected in the anterior control region (data not shown). Therefore, the growth arrest phenotype of early *scrib* mutant tumors is unlikely to be caused by increased apoptosis. The cell volume of individual *scrib* mutant cells was larger than that of the control wing disc cells (Fig. S3). Therefore, the growth arrest of early *scrib* mutant tumors is also unlikely to be caused by defects in the growth of individual cells.

We then performed flow cytometry analysis with wild-type imaginal discs and *scrib* mutant tumors expressing fluorescence ubiquitin cell cycle indicator (FUCCI) sensors to examine possible cell proliferation defects (Zielke et al., 2014). In wild-type discs at day 4 AEL, about 60% of cells were in the G0/G1 phase, 25% in the S phase and 15% in the G2/M phase (Fig. 1D,G), in agreement with previous reports (Milan et al., 1996). However, in *scrib* mutant tumors at 4 days AEL about 25% of cells were in the G0/G1 phase, 25% in the S phase and 50% in the G2/M phase (Fig. 1E,G). The results suggest that a population of cells in early *scrib* mutant tumors is arrested at the G2/M stage.

*Drosophila* larval brain neuroblasts are an excellent model for analyzing mitosis because of their accessibility for live imaging (Cabernard and Doe, 2013). We observed that *scrib* mutant neuroblasts frequently displayed a significantly prolonged entry into mitosis (Fig. S4), consistent with the G2/M arrest defects observed in early *scrib* mutant wing disc cells. Notably, by day 8 AEL, the cell cycle distribution of *scrib* mutant cells was comparable with that of day 4 wild-type imaginal discs (Fig. 1F,G). Therefore, the growth arrest phenotype in early *scrib* mutant tumors is caused by cell cycle defects, which can resolve over time.

### The *scrib* mutant tumors from different time points display distinctive global transcriptomic signatures

**Fig. 2.**
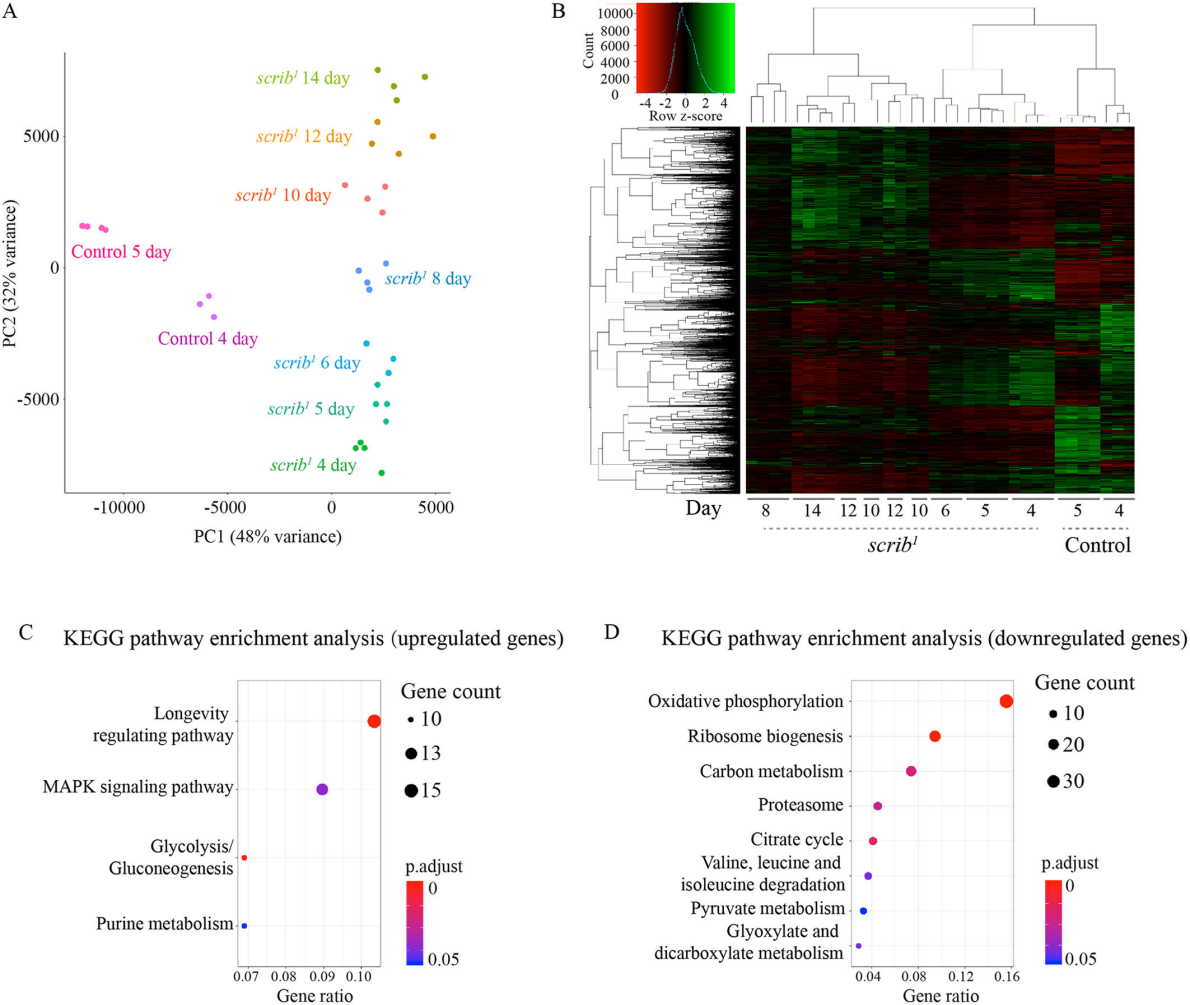
**The *scrib* mutant tumors from different stages display distinctive global transcriptomic signatures.** (A) PCA of transcriptomes from control and *scrib* mutant wing imaginal discs collected at different ages. Control genotype was *FRT82B*. Three or four biological replicates were plotted for each time point. Low-expression genes were filtered using a cutoff of baseMean>100 calculated in DEseq2. A matrix of 6901 gene counts×34 samples was used to compute PCA after gene counts were transformed using the default DEseq2 rlogTransformation function. (B) Hierarchical clustering of transcriptomes from control and *scrib* mutant wing imaginal discs collected at different ages. Control genotype was *FRT82B*. Three or four biological replicates were plotted for each time point. Gene filtering criteria were the same as for A. A matrix of 6901 gene counts×34 samples was used for hierarchical clustering after gene counts were transformed to counts per million (cpm) value using the edgeR cpm function and scaled using the scale function in R. (C,D) KEGG pathway enrichment analysis for the top 10% of upregulated genes (C) and the bottom 10% of downregulated genes (D) over time with a cutoff of adjusted *P*-value <0.05. The pathway enrichment analysis was performed using enrichKEGG function in the clusterProfiler package.

Early and late *scrib* mutant tumors display different growth rates and cell cycle profiles, indicative of potential evolving capacity. We therefore performed bulk RNA sequencing (RNA-seq) for wild-type imaginal discs and *scrib* mutant tumors collected at different time points to gain a global view of transcriptomic changes associated with *scrib* tumor progression (Fig. 2). The biological repeats were well clustered per sample for each time point and the *scrib* mutant tumors formed a transition trajectory along time on the principal component analysis (PCA) plot (Fig. 2A). Both PCA and hierarchical clustering analyses suggested that the *scrib* mutant tumors could be grouped into three stages with distinctive transcriptomic signatures: an early stage at 4-6 days AEL, an intermediate stage at 8 days and a late stage at 10-14 days (Fig. 2A,B).

We performed linear regression analysis for each gene in the *scrib* tumor time series and ranked the genes by the slope of the regression line (Fig. S5). For the 10% of genes ranked at the top and bottom, we performed pathway enrichment analysis with the KEGG pathway database (Kyoto Encyclopedia of Genes and Genomes, https://www.genome.jp/kegg/) (Fig. 2C,D) as well as the *Drosophila* GLAD database (Gene List Annotation for *Drosophila*, https://www.flyrnai.org/tools/glad/web/) (Hu et al., 2015) (Fig. S6). For example, the expression level of genes related to oxidative phosphorylation decreased over time and the expression level of glycolytic genes increased over time in the *scrib* mutant tumors, consistent with a glucose metabolism drift widely observed in tumors called the Warburg effect (Eichenlaub et al., 2018; Wang et al., 2016). Of the several conserved signaling pathways that show significant changes in *scrib* mutant tumors over time, the pathway enrichment analysis with both KEGG and GLAD databases pointed us to the MAPK signaling pathway (Fig. 2C; Fig. S6A). Notably, two of the three *Drosophila* MAPK pathway subgroups, the JNK and ERK pathways, have long been known as crucial for regulating the growth outcome of *scrib* mutant mosaic clones (Brumby and Richardson, 2003; Cordero et al., 2010; Igaki et al., 2006; Pagliarini and Xu, 2003; Uhlirova and Bohmann, 2006). Therefore, although the transcriptomic analysis suggested that *scrib* mutant tumors have extensive changes besides the MAPK pathway, including several metabolic pathways and other signaling pathways such as the Notch and JAK/STAT pathways, we first focused on examining whether JNK and ERK signaling activities change over time and whether these changes have functional implications regarding *scrib* tumor growth.

### High JNK signaling activity inhibits proliferation in the early *scrib* mutant tumors

**Fig. 3.**
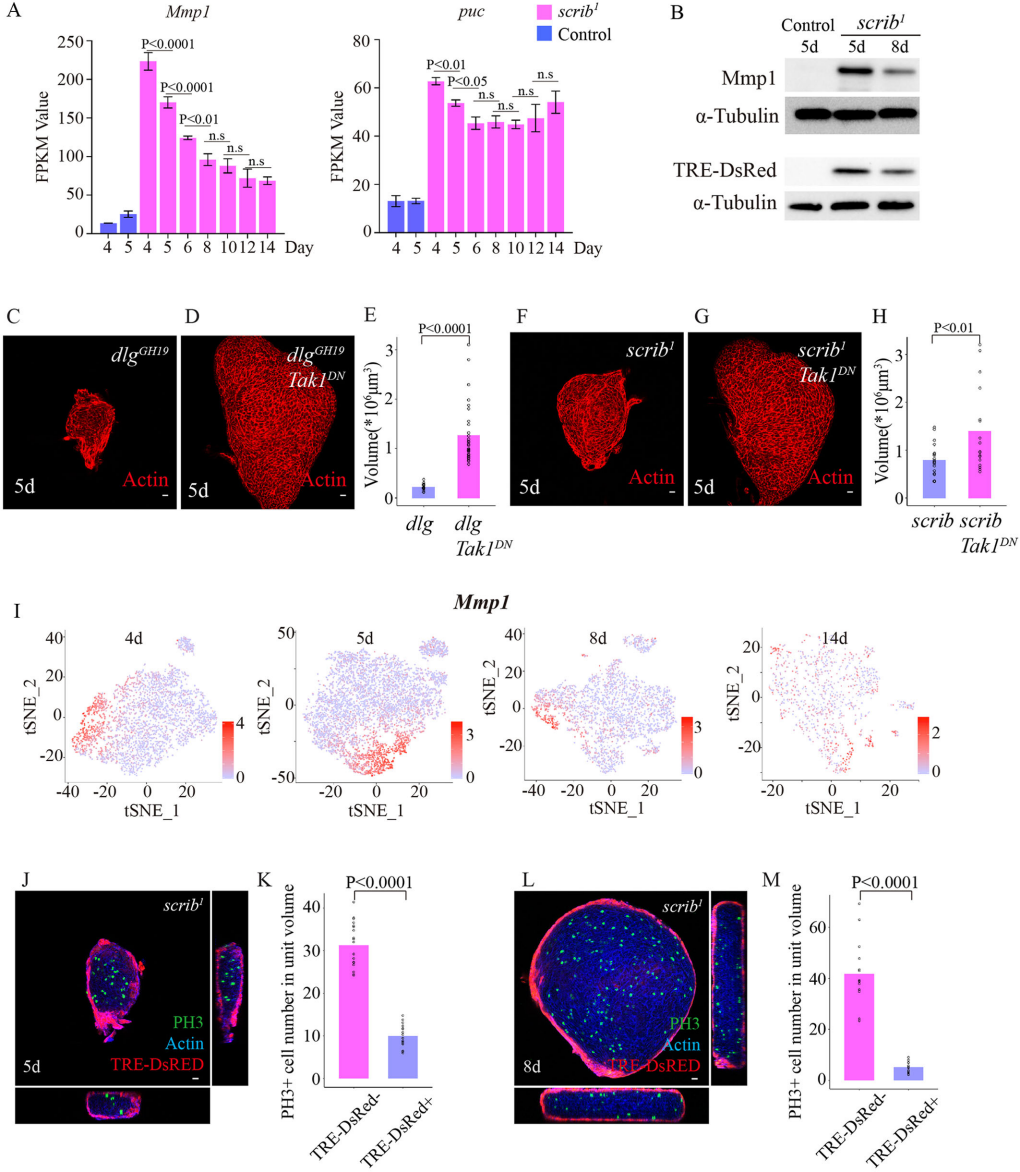
**High JNK signaling activity causes growth arrest in early *scrib* mutant tumors.** (A) Gene expression levels for *puc* and *Mmp1*, the JNK signaling activity reporter genes, as FPKM (fragments per kilobase million) values measured in the bulk RNA-seq experiments shown in Fig. 2A,B. Control genotype was *FRT82B*. Statistical analysis was performed using the one-way ANOVA test. (B) Western blot analysis of Mmp-1 and TRE-DsRed protein levels in control imaginal discs at 5 days AEL and *scrib^1^* mutant wing imaginal discs at 5 and 8 days AEL. Control genotype was *scrib^1^/TM6B*. (C,D) Imaginal discs from *dlg^GH19^* (C) and *dlg^GH19^ Tak1^DN^* (D) at 5 days AEL, stained for actin (red). Genotype for C was *dlg^GH19^*/Y; *c855a-Gal4/*^+^, *n*=19, 2.2±0.6×10^5^ μm^3^. Genotype for D was *dlg^GH19^*/Y; UAS-*Tak1^DN^/^+^; c855a-Gal4/^+^, n*=30, 1.3±0.6×10^6^ μm^3^. (E) Quantification of the tumor volumes. Statistical analysis was performed by unpaired *t*-test. (F,G) Imaginal disc from *scrib^1^* (F) and *scrib^1^ Tak1^DN^* (G) at 5 days AEL, stained for actin (red). Genotype for F was *c855a-Gal4 scrib^1^/scrib^1^*, *n*=19, 8±3×10^5^ μm^3^. Genotype for G was UAS-*Tak1^DN^/^+^; c855a-Gal4 scrib^1^/scrib^1^*, *n*=17, 1.4±0.9×10^6^ μm^3^. (H) Quantification of the tumor volumes. Statistical analysis was performed by unpaired *t*-test. (I) t-SNE projection of *scrib^1^* mutant cells at 4, 5, 8 and 14 days AEL, where individual cells are colored by the normalized expression level of *Mmp1*. The gene expression level normalization was performed using the default LogNormalize method in Seurat. (J,L) AEL *scrib^1^* imaginal disc at 5 (J) and 8 (L) days AEL, stained for actin (blue) and PH3 (green). Genotype was TRE-dsRED/^+^; *scrib^1^/scrib^1^*. (K,M) Quantification of the PH3^+^ cell number per unit volume. 5 days TRE-DsRed^–^, *n*=20, 3.1±0.5/10^8^ μm^3^; 5 days TRE-DsRed^+^, *n*=20, 10±2/10^7^ μm^3^. 8 days TRE-DsRed^–^, *n*=15, 4±1/10^8^ μm^3^; 8 days TRE-DsRed^+^, *n*=15, 5±2/10^7^ μm^3^. Statistical analysis was performed by unpaired *t*-test. Scale bars: 10 μm.

We first examined JNK signaling activity because the expression levels of two widely used JNK signaling target genes, *Mmp1* and *puckered* (*puc*) (Igaki et al., 2006; Uhlirova and Bohmann, 2006), as measured in the bulk RNA-seq experiments (Fig. 2), decreased significantly in *scrib* mutant tumors over time (Fig. 3A). Although JNK signaling activity is elevated in *scrib* tumors of all stages compared with wild-type wing discs (Fig. 3A,B), in agreement with previous reports (Bunker et al., 2015; Igaki et al., 2006), we found that the protein levels of Mmp1 and TRE-DsRED, a JNK signaling reporter (Chatterjee and Bohmann, 2012), decreased significantly in *scrib* tumors at 8 days AEL compared with *scrib* tumors at 5 days AEL (Fig. 3B). These data suggest that JNK signaling activity decreases as the *scrib* tumors grow over time.

We also found that the growth arrest phenotype in early *scrib* or *dlg* tumors was effectively rescued through overexpression of a dominant-negative form of Tak1 (Tak1^DN^) or Basket (Bsk^DN^), which block JNK signaling activity (Fig. 3C-H; Fig. S7). Notably, overexpression of Ras^V12^, NICD or Yki^S168A^ (an active form of Yki), which are known to promote *scrib* clonal growth in a mosaic setting (Brumby and Richardson, 2003; Chen et al., 2012; Pagliarini and Xu, 2003), did not rescue the growth arrest phenotype in early *scrib* tumors (Fig. S8). Note that we used a C855a-Gal4 driver that expresses early in whole *scrib* or *dlg* tumors and does not cause lethality when Ras^V12^ or NICD are co-expressed (Fig. S9). Together, the data suggest that high JNK activity is a potent inhibitor of tumor growth in early *scrib* tumors. This result is consistent with a recent study showing that injury-induced JNK activation in wing imaginal discs also leads to cell cycle arrest at G2 (Cosolo et al., 2019).

We next examined why JNK signaling activity decreases as the *scrib* tumors grow. From single-cell transcriptomic data generated from *scrib* mutant tumors, we found that expression of the JNK signaling reporter gene *Mmp1* is highly heterogeneous (Fig. 3I). This data led us to re-examine the Mmp1 expression pattern; we found that Mmp1 showed strongest signals at the periphery of *scrib* mutant tumors (Movies 1, 2). To exclude the possibility that the Mmp1 staining pattern was an artifact from poor antibody penetrance, we examined the expression pattern of a JNK signaling reporter TRE-DsRED without using antibody staining. Similarly, we found that TRE-dsRED displayed a radial polarity, with the strongest fluorescence signals at the periphery of *scrib* mutant tumors (Fig. 3J-M). Moreover, single-cell transcriptomic analysis demonstrated that *Mmp1* expression shows high correlation with a number of cytoskeletal genes, such as *cindr* (Table S1; Fig. S10). A protein-trap line of *cindr* also revealed a radial polarity in *scrib* mutant tumors (Movies 3, 4). Therefore, JNK signaling activation in homozygous *scrib* mutant tumors exhibited a radial polarity, with JNK^high^ cells located at the surface of the *scrib* tumors. Because *scrib* mutant tumors grow in a compact manner, the surface-to-volume ratio drops as the tumor volumes increases, leading to a decrease in the percentage of JNK^high^ G2/M-arrested cells over time.

### ERK signaling activity increases over time and promotes tumor growth in late-stage *scrib* mutant tumors

**Fig. 4.**
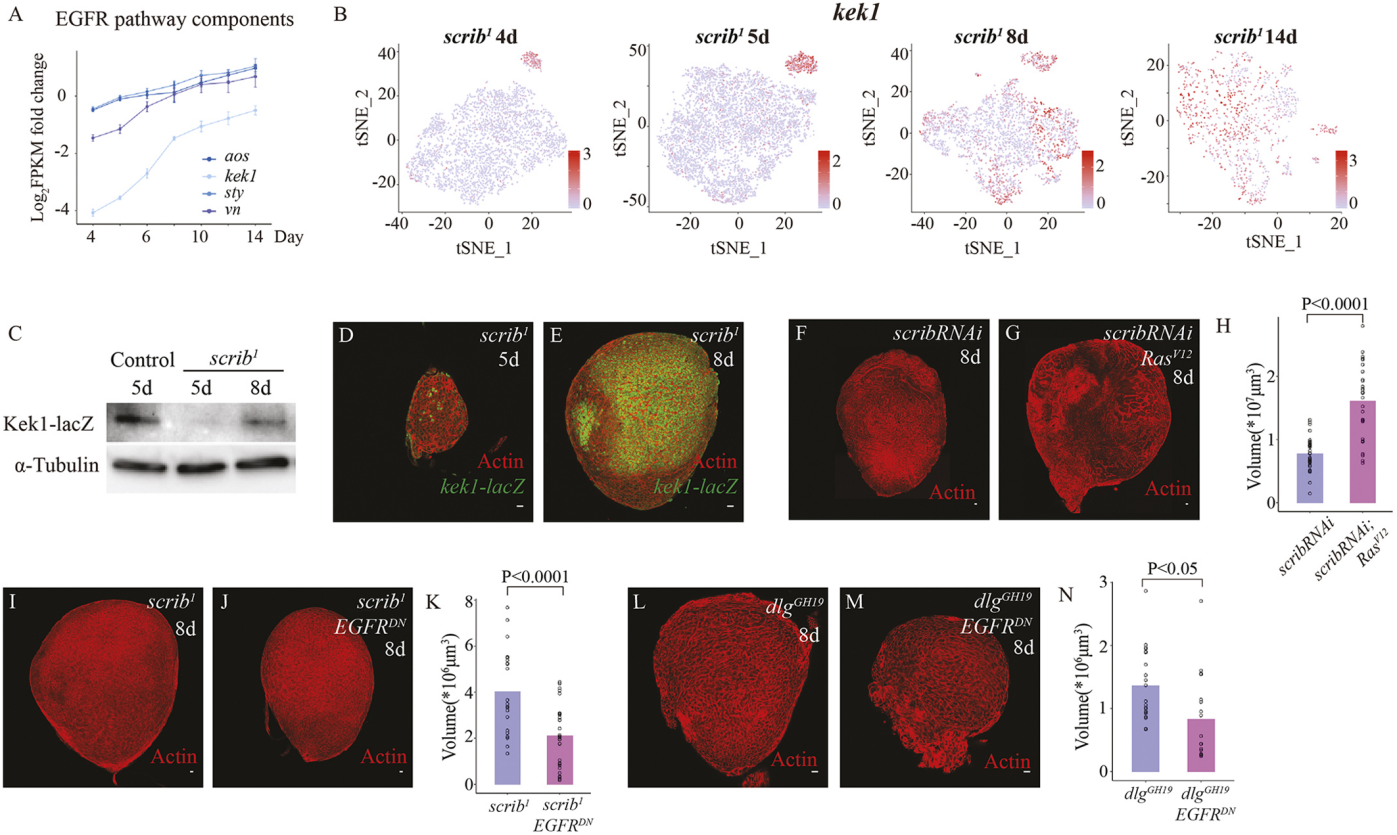
**ERK signaling activity increases over time and promotes growth of late-stage *scrib* mutant tumors.** (A) Expression levels for *aos*, *kek1*, *sty*, the EGFR signaling activity reporter genes, and *vn*, an EGFR signaling ligand, in *scrib* mutant tumors normalized by the respective expression level in control imaginal discs at 5 days AEL using log_2_(FPKM fold change) values. Control genotype was *FRT82B*. (B) t-SNE projection of *scrib^1^* mutant cells at 4, 5, 8 and 14 days AEL, where individual cells are colored by the normalized expression level of *kek1*. The gene expression level normalization was performed using the default LogNormalize method in Seurat. (C) Western blot analysis of β-galactosidase protein level in control imaginal discs at 5 days AEL and in *kek1-lacZ*; *scrib^1^* mutant wing imaginal discs at 5 and 8 days AEL. Control genotype was *kek1-lacZ/^+^; scrib^1^/TM6B*. (D,E) *kek1-lacZ*; *scrib^1^* mutant tumors at 5 (D) and 8 (E) days AEL, stained for β-galactosidase (green) and actin (red). (F,G) *scrib RNAi* (F) and *scrib RNAi Ras^V12^*(G) imaginal discs at 8 days AEL, stained for actin (red). Genotype for F was *c855a-Gal4/UAS-scrib RNAi, n*=32, 8±3×10^6^ μm^3^. Genotype for G was UAS-*Ras^V12^/^+^; c855a-Gal4/UAS-scrib RNAi*, *n*=28, 1.6±0.7×10^7^ μm^3^. (H) Quantification of the tumor volumes. (I,J) *scrib^1^* (I) and *scrib^1^ EGFR^DN^* (J) imaginal discs at 8 days AEL, stained for actin (red). Genotype for (I) was *c855a-Gal4 scrib^1^/scrib^1^, n*=21, 4±2×10^6^ μm^3^. Genotype for J was UAS-*EGFR^DN^/^+^; c855a-Gal4 scrib^1^/ scrib^1^*, *n*=31, 2±1×10^6^ μm^3^. (K) Quantification of the tumor volumes. (L,M) *dlg^GH19^* (L) and *dlg^GH19^ EGFR^DN^* (M) imaginal discs at 8 days AEL, stained for actin (red). Genotype for L was *dlg^GH19^*/Y; *c855a-Gal4/*^+^, *n*=21, 1.4±0.6×10^6^ μm^3^. Genotype for M was *dlg^GH19^*/Y; UAS-*EGFR^DN^/^+^; c855a-Gal4/* UAS-*EGFR^DN^*, *n*=18, 8±7×10^5^ μm^3^. (N) Quantification of the tumor volumes. All statistical analyses were performed by unpaired *t*-test. Scale bars: 10 μm.

The bulk *scrib* tumor time series transcriptomic data demonstrated that components of the ERK signaling pathway also showed significant changes (Fig. 4A). Moreover, when we ordered genes by the change in the percentage of cells expressing the specific gene in single-cell transcriptomic data, we noticed that *kekkon1 (kek1)*, a well-established ERK signaling target (Ghiglione et al., 2003, 1999), was ranked at the top. Few *kek1^+^* cells existed in early *scrib* tumors and the majority of cells were *kek1^+^* in late *scrib* tumors (Fig. 4B; Fig. S11). Using a *kek1-lacZ* line (Musacchio and Perrimon, 1996), we confirmed that late *scrib* tumors indeed harbored many more *kek1^+^* cells than early *scrib* tumors (Fig. 4C-E). Similarly, the expression of *sprouty (sty)* (Casci et al., 1999) and *argos (aos)* (Golembo et al., 1996), two other genes induced by ERK activity, also increased over time (Fig. 4A; Fig. S12). Moreover, the expression of *vein* (*vn)*, an EGFR ligand promoting patterning and proliferation in wing imaginal discs (Schnepp et al., 1996), also increased over time (Fig. 4A; Fig. S12). Together, these data suggest that ERK activity increases over time as *scrib* mutant tumors grow.

ERK signaling activity is required for both growth and patterning in wing imaginal discs (Diaz-Benjumea and Hafen, 1994; Prober and Edgar, 2000; Zecca and Struhl, 2002a,b). Here, we focused on examining whether increasing ERK activity over time influences *scrib* tumor growth. Interestingly, although overexpression of Ras^V12^ did not rescue early *scrib* mutant tumors from growth arrest (Fig. S8), it significantly increased the size of *scrib* mutant tumors at later stages (Fig. 4F-H). Conversely, blocking ERK signaling activity through expression of a dominant-negative form of EGFR (Buff et al., 1998) (Fig. 4I-N) and ERK and Ras RNAi constructs (Fig. S13) led to a reduction in *scrib* and *dlg* mutant tumor sizes at later stages. These data suggest that an increase in ERK signaling activity promotes *scrib* tumor growth at later stages.

Interestingly, ERK and JNK pathways have extensive cross-talks in *scrib* mutant tumors, as indicated by the high correlation of a number of ERK pathway components with Mmp1 expression at the single-cell level (Table S1; Fig. S14). Moreover, when we blocked JNK signaling activity in early *scrib* mutant tumors, we observed an increase in *kek1^+^* cell number (Movies 5, 6; Fig. S15) in addition to an increase in tumor size (Fig. 3C-H), indicating that high JNK signaling activity possibly represses ERK activity in early *scrib* mutants. However, the molecular mechanism mediating JNK repression of ERK activity remains unclear.

## DISCUSSION

Here we have demonstrated that dynamic changes in JNK and ERK activities underlie a transition from a growth arrest state to a proliferation state over time in *Drosophila scrib* mutant tumors.

We found that JNK signaling activation exhibited a radial polarity, with JNK^high^ cells located at the surface of homozygous *scrib* mutant tumors. We do not yet know the underlying reason for the heterogeneous JNK activation pattern. One possible reason is that the active JNK ligand Eiger might be mostly provided from external sources. This might involve hemocytes (Cordero et al., 2010) or the fat body, as recently shown for another nTSG mutant *alg3* (de Vreede et al., 2018). Another possible input is potential mechanical stresses that the tumor surface cells experience because JNK signaling is activated at leading-edge cells, which assemble supracellular actin cables and experience high actomyosin-dependent tensile force during dorsal closure (Martin-Blanco et al., 1998).

JNK activation induced by injury in the wing imaginal discs also leads to cell cycle arrest at G2, which can be restored by overexpression of Cdc25/String (Cosolo et al., 2019). We found that expression of *Cdc25*/*string(stg)* and *Cks30A* anti-correlates with *Mmp1* in the single-cell data. Moreover, we could not rescue growth arrest in early *scrib* mutant tumors by overexpression of String alone (data not shown), indicating that the cell cycle defects in early *scrib* mutant tumors are probably mediated by multiple factors downstream of high JNK signaling activity.

Clonal *scrib* mutant cells are eliminated through cell competition when they are surrounded by wild-type neighbors (Brumby and Richardson, 2003; Vaughen and Igaki, 2016; Yamamoto et al., 2017). It is noteworthy that clonal *scrib* mutant cells are likely to be in a different state from growth-arrested homozygous *scrib* mutant cells. Studies have shown that overexpression of Ras^V12^, NICD and p35 can effectively block the clonal *scrib* mutant cells from undergoing apoptosis induced by cell competition. In our study, we found that overexpression of Ras^V12^, NICD and p35 had little effect in relieving the early *scrib* mutant tumors from growth arrest. It is likely that interactions between clonal *scrib* mutant cells and wild-type cells during cell competition induce an additional layer of complexity into determination of clonal *scrib* mutant cell state, as the effects of JNK activation are known to be highly context dependent (Pinal et al., 2019).

In human solid tumors, the tumor margin and core were also shown to experience different immune environments (Kather et al., 2018). Our study highlights that tissue topological factors (peripheral versus center) can be an inherent source of diversity in cell populations in growing tumors and that dynamic signaling rewiring during the processes of early tumorigenesis does not necessarily require the generation of *de novo* mutations or new cell clones.

## MATERIALS AND METHODS

### Fly stocks

The fly strains used in this study were *scrib^1^* FRT82B/TM6B (Bilder et al., 2000), *dlg^GH19^* FRT19A/FM7c (a kind gift from the Schupbach laboratory, Princeton University; a C-to-T mutation led to a stop codon at Q42), UAS-*scrib* RNAi on the second chromosome (Bloomington/BL38199), UAS-*scrib* RNAi on the third chromosome (BL35748), UAS-*dlg* RNAi on the third chromosome (BL35772), UAS-Ras85D RNAi (BL34619), UAS-ERK RNAi(BL34855), y[1] v[1]; P{y[+t7.7]=CaryP}attP2 (BL36303, the third chromosome TRiP line background strain), y[1] v[1]; P{y[+t7.7]=CaryP}attP40 (BL36304, the second chromosome TRiP line background strain), engrailed-Gal4 UAS-GFP (BL25752), engrailed-Gal4 (BL30564), UAS-p35 (Hay et al., 1994) (BL6298), Fly-FUCCI (BL55098), worGal4, UAS-cherry::Jupiter, Sqh::GFP (Cabernard and Doe, 2013), c855a-Gal4 (Hrdlicka et al., 2002) (BL6990), UAS-Ras^V12^ (Karim and Rubin, 1998), UAS-Yki^S168A^ (Oh and Irvine, 2009) (BL28818), UAS-NICD (Rebay et al., 1993), UAS-Bsk^DN^ (Igaki et al., 2006) (a kind gift from Jose C. Pastor-Pareja School of Life Sciences, Tsinghua University, China), UAS-Tak1^DN^ (BL58811), TRE-dsRED (Chatterjee and Bohmann, 2012), kek1-lacZ (Ghiglione et al., 2003) and UAS-EGFR^DN^ (Buff et al., 1998) (BL5364), cindr^CA06686^ (BL50802).

### Immunohistochemistry

About 50 embryos collected within 3 h were put into an individual vial of fly food to avoid crowding and the larvae raised in an incubator at 25°C for appropriate lengths of time before dissection. Imaginal discs were fixed and stained according to standard protocols. The primary antibodies used were mouse anti-phospho-Histone3 (1:1000; Cell Signaling Technology), mouse anti-Mmp 1(1:30; a mixture of 5H7B11, 3A6B4 and 3B8D12, DSHB) and mouse anti-beta-galactosidase (1: 25, 40-1a, DHSB). The secondary antibodies conjugated with various Alexa Fluor dyes (Thermo Fisher Scientific) were used at 1:500. Phalloidin conjugated with Alexa Fluor dyes (1:1000, Thermo Fisher Scientific) and Hoechst 33342 (1:10,000, Thermo Fisher Scientific) were used to stain F-actin and DNA, respectively. All images were acquired on a Leica TCS SP8 confocal microscope.

### Western blotting

About 30 larvae were dissected in PBS. Cell lysates were homogenized in 1× RIPA (Millipore) with protease inhibitors (Roche). The primary antibodies used were mouse anti-MMP1 (1:100; a mixture of 5H7B11, 3A6B4 and 3B8D12, DSHB), rabbit anti-DsRed (1:1000; Takara) mouse anti-beta-galactosidase (1:250; 40-1a, DHSB) and mouse anti-alpha-tubulin (1:5000; 12G10, DSHB).

### Image processing and data analysis

Images were taking as z-stacks with a step size of 1 μm. Tissue volume was measured with Measure Stack plugin in Fiji using the actin-staining channel; the wand tool was used to select regions of interest, where manual adjustment of tolerance value and recognition of edges were made for each slice. PH3^+^ cell number was calculated with the Cell Counter plugin in Fiji.

### Fluorescence-activated cell-sorting

Wing imaginal discs were dissociated in 0.25% trypsin-EDTA solution at 37°C for 10 min and stained with Hoechst at 37°C for 45 min. Cells were sorted with BDFACSAria IIIu and the data analyzed with FlowJo.

### *Drosophila* neuroblast live imaging

Female virgins of hsFLP; worGal4, Sqh::GFP,UASCherry::jupiter; FRT82B/TM6B were crossed with males of *scrib*FRT82B/TM6B. Progeny were heat-shocked at 38°C (in the water bath) for 1 h and subsequently raised at 25°C until imaging. Larvae (5 days AEL) were then dissected and imaged according to standard protocols (Cabernard and Doe, 2013). For wild-type controls, larvae expressing wt; worGal4,Sqh::GFP,UASCherry::jupiter; Dr/TM6B were dissected and imaged with the same laser setting as that of the *scrib* mutant neuroblasts.

### Bulk RNA-seq and data analysis

Total RNA was extracted from control and *scrib^1^* wing imaginal discs with the RNeasy Mini Kit (Qiagen). Construction of cDNA libraries and 150 bp paired-end sequencing on Illumina HiSeq platform were performed by Novogene. Cleaned raw reads obtained from Novogene were mapped to the reference genome (BDGP6) using STAR (2.5.4a) (Dobin et al., 2013). Default parameters were used except for the max intron size, which was set at 100,000. Counts were generated by featureCounts available in Subread package (1.32.2) with the default setting (Liao et al., 2013). PCA analysis was performed in DESeq2 (1.22.2) after low expression genes were filtered (baseMean>100) (Love et al., 2014). Count normalization to counts per million (cpm) was performed using edgeR (3.24.3) before hierarchical clustering (hclust function in R) (Robinson et al., 2010). Linear regression was performed using the lm function in R (3.5.2). Pathway enrichment analysis was performed using the default setting of clusterProfiler (3.10.1) (Yu et al., 2012). The GEO accession number for these data is GSE130243.

### 10x Genomics single cell RNA-seq data analysis

Single-cell RNA-seq data from staged *scrib^1^* wing imaginal discs were generated in another study (M.D., Y.W., L.Z., Yang Yang, S.H., J.W., Hao Ge, Toyotaka Ishibashi and Y.Y., unpublished; GEO accession number GSE130566). Raw data mapping and primary analysis was performed in the Cell Ranger pipeline. The t-SNE plot for marker gene expression pattern was plotted using Seurat (v2.3.4) using default settings (Butler et al., 2018). To calculate the correlation coefficients for any two genes *x* and *y* in single cells, the normalized expression levels of genes *x* and *y* form two numeric vectors that can be used to compute the correlation coefficient (*r*) using Pearson’s formula:
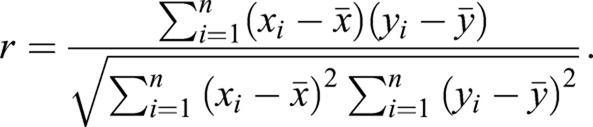


## Supplementary Material

Supplementary information
