# Supplementary material for: Dynamic MAPK signaling activity underlies a transition from growth arrest to proliferation in Drosophila scribble mutant tumors
Source: Dis Model Mech. 2019 Aug 29;12(8):dmm040147. doi: 10.1242/dmm.040147 (PMC6737955; doi:10.1242/dmm.040147)
Supplement: Supplementary information [file dmm-12-040147-s1.pdf]

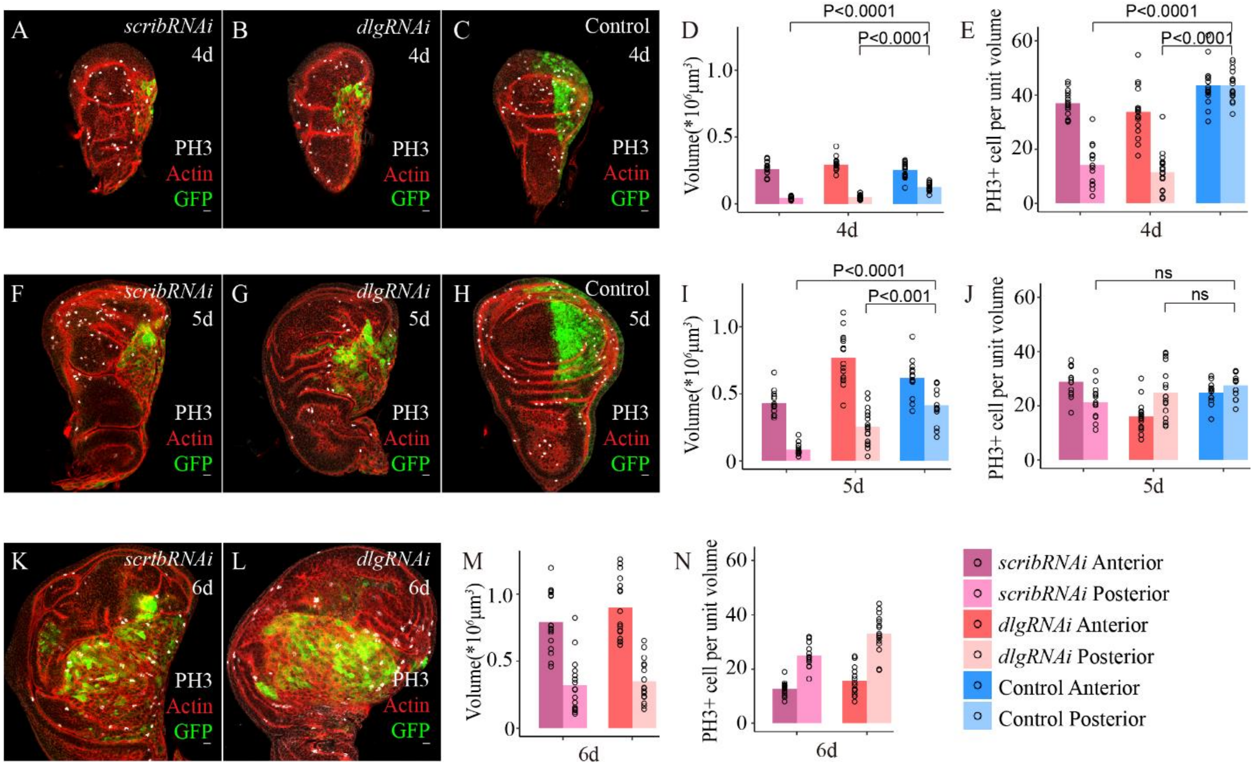

Figure S1. The *scrib* RNAi and *dlg* RNAi cells in the wing disc posterior domain change from a growth arrest state to a proliferative state.

4-day (A-C), 5-day (F-H) and 6-day (K-L) AEL *scrib RNAi* (A, F, K), *dlg RNAi* (B, G, L) and control (C, H) imaginal discs stained for PH3 (gray), actin(red) and GFP (green). Quantification of volumes (D, I, M) and PH3+ cell number per unit volume ($10^5\mu m^3$) (E, J, N) for 4-day (D, E), 5-day (I, J) and 6-day (M, N) AEL *scrib RNAi*, *dlg RNAi* and control imaginal discs. Genotype for the *scrib RNAi* group: *engrailed-Gal4 UAS-GFP/+; UAS-scribRNAi/+*. Genotype for the *dlg RNAi* group: *engrailed-Gal4 UAS-GFP/+; UAS-dlgRNAi/+*. Genotype for the control group: *engrailed-Gal4 UAS-GFP/+; P{y[+t7.7]=CaryP}attP2 /+* (the 3rd chromosome TRiP line background strain). The *scrib RNAi* group, **4d** n = 15, anterior, $2.6\pm0.5\times10^5\mu m^3$, PH+ cell number 37±5, posterior, $4\pm1\times10^4\mu m^3$, PH+ cell number 14±8, **5d** n = 14, anterior, $4.3\pm0.9\times10^5\mu m^3$, PH+ cell number 29±5, posterior, $8\pm4\times10^4\mu m^3$, PH+ cell number 21±6, **6d** n = 15, anterior, $8\pm2\times10^5\mu m^3$, PH+ cell number 13±3, posterior, $3\pm2\times10^5\mu m^3$, PH+ cell number 25±4; The *dlg RNAi* group, **4d** n = 16, anterior, $3.0\pm0.5\times10^5\mu m^3$, PH+ cell number 34±9,

posterior, $5\pm2\times10^{4}\mu m^{3}$, PH+ cell number 11±8, **5d** n = 17, anterior, $8\pm2\times10^{5}\mu m^{3}$, PH+ cell number 16±5, posterior, $3\pm1\times10^{5}\mu m^{3}$, PH+ cell number 25±9, **6d** n = 16, anterior, $9\pm2\times10^{5}\mu m^{3}$, PH+ cell number 16±5, posterior, $4\pm2\times10^{5}\mu m^{3}$, PH+ cell number 33±7; The control group, **4d** n = 16, anterior, $2.5\pm0.6\times10^{5}\mu m^{3}$, PH+ cell number 44±8, posterior, $1.3\pm0.3\times10^{5}\mu m^{3}$, PH+ cell number 43±6, **5d** n = 13, anterior, $6\pm1\times10^{5}\mu m^{3}$, PH+ cell number 25±4, posterior, $4\pm1\times10^{5}\mu m^{3}$, PH+ cell number 28±4. Scale bar: 10μm. Statistical analysis was performed by unpaired t-test. Note that larvae from the control group become pupae at 5-day AEL.

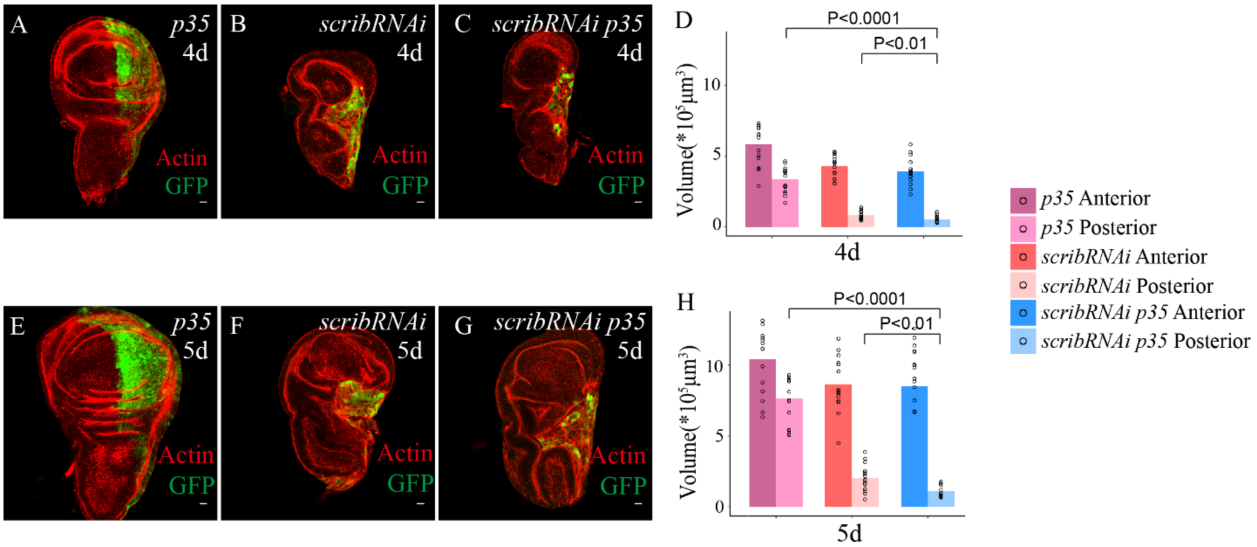

Figure S2. Overexpression of p35 does not rescue the growth arrest phenotype in the *scrib* RNAi cells.

4-day (A-C) and 5-day (E-G) AEL *p35* (A, E), *scrib RNAi* (B, F) and *scrib RNAi p35* (C, G) imaginal discs stained for actin (red) and GFP (green). Quantification of volumes (D, H) for 4-day (D) and 5-day (H) AEL *p35*, *scrib RNAi* and *scrib RNAi p35* imaginal discs. Genotype for the *p35* group: *UAS-p35/+; engrailed-Gal4 UAS-GFP/+*. Genotype for the *scrib RNAi* group: *engrailed-Gal4 UAS-GFP/UAS-scribRNAi*. Genotype for the *scrib RNAi p35* group: *UAS-p35/+; engrailed-Gal4 UAS-GFP/UAS-scribRNAi*. *p35*, **4d** n = 15, anterior, $6\pm1\text{x}10^5\mu m^3$, posterior, $3.4\pm0.9\text{x}10^5\mu m^3$, **5d** n = 15, anterior, $1.0\pm0.2\text{x}10^6\mu m^3$, posterior, $8\pm2\text{x}10^5\mu m^3$; *scrib RNAi*, **4d** n = 16, anterior, $4.3\pm0.8\text{x}10^5\mu m^3$, posterior, $8\pm3\text{x}10^4\mu m^3$, **5d** n = 15, anterior, $9\pm2\text{x}10^5\mu m^3$, posterior, $2.0\pm0.9\text{x}10^5\mu m^3$; *scrib RNAi p35*, **4d** n = 15, anterior, $4\pm1\text{x}10^5\mu m^3$, posterior, $5\pm2\text{x}10^4\mu m^3$, **5d** n = 15, anterior, $8\pm2\text{x}10^5\mu m^3$, posterior, $1.1\pm0.4\text{x}10^5\mu m^3$. Statistical analysis was performed by unpaired t-test. Scale bar: 10µm.

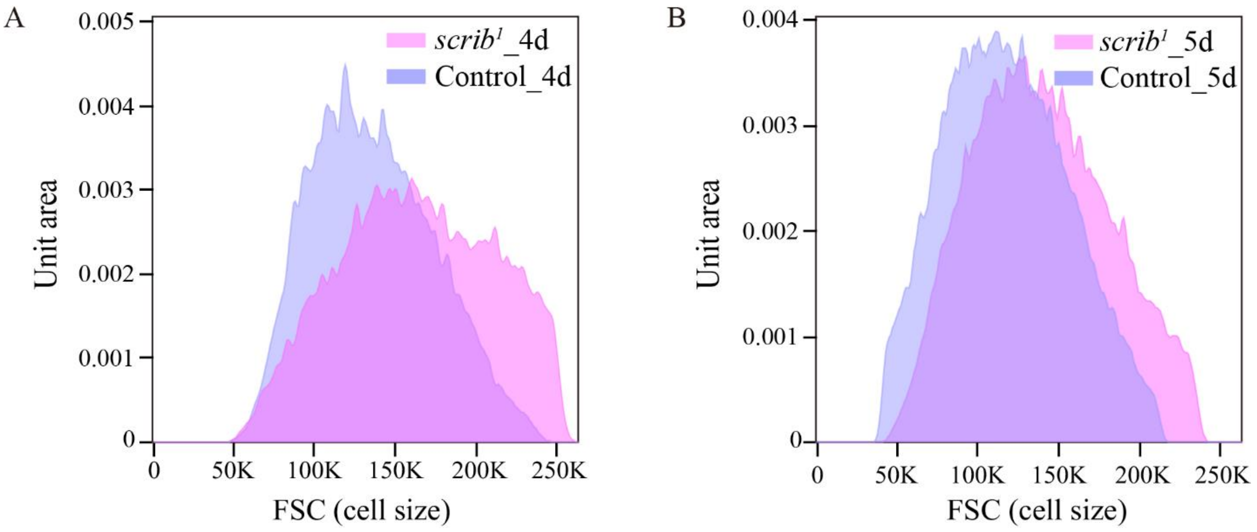

Figure S3. The *scrib* mutant cells do not show defects in individual cell growth.

(A-B) FACS analysis of cell size for control and *scrib*^{1} mutant cells from 4-day (A) and 5-day (B) AEL wing imaginal discs. Genotype for the control group: *FRT82B*. At least 10,000 cells were recorded for each cell group.

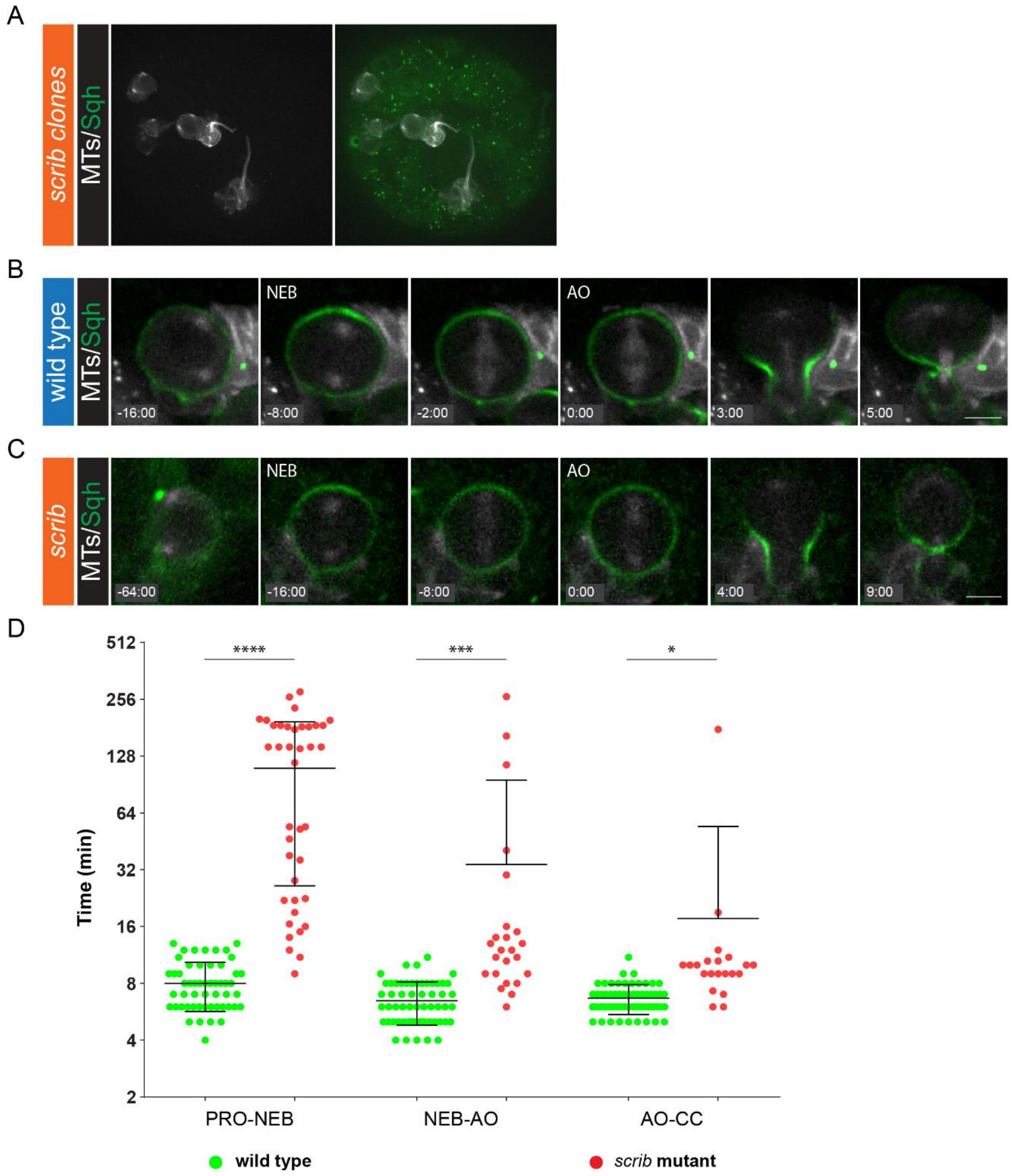

Figure S4. The *scrib* mutant neuroblasts prolong mitosis.

(A) Representative *scrib* mutant clones co-expressing the spindle marker (Cherry::jupiter; white) and non-muscle Myosin II (Sqh::GFP; green). Representative image sequences of a (B) wild type, and (C, D) *scrib* mutant neuroblasts expressing the clonal marker Cherry::jupiter (white)

Disease Models & Mechanisms • Supplementary information

and non-muscle Myosin II (Sqh::GFP; green). Nuclear envelope breakdown (NEB) and anaphase onset (AO) were used to define mitotic stages. "0:00" indicates AO. (D) Comparison of the cell cycle length at various stages during mitosis between wild type neuroblasts (n = 58) and the *scrib* mutant neuroblasts (n = 44). PRO-NEB: Prophase - Nuclear envelope breakdown. NEB – AO: Nuclear envelope breakdown to anaphase onset. AO – CC: Anaphase onset to Completion of cytokinesis. Scale bar: 5 µm. Asterisk denote statistical significance, derived from unpaired t-tests: *; $p \leq 0.05$, **; $p \leq 0.01$, ***; $p \leq 0.001$, ****; $P \leq 0.0001$, n.s.; not significant.

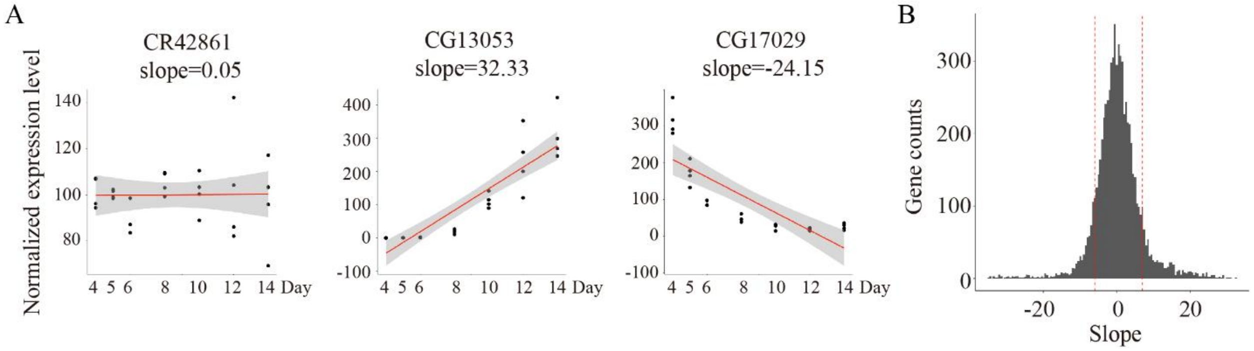

Figure S5. Linear regression analysis of gene expression temporal profiles identifies genes with the most significant changes in the *scrib* mutant tumors over time.

(A) Plot of exemplary gene expression temporal profiles representing three categories of genes, the expression of which does not change (CR42861), increases (CG13053) and decreases (CG17029) over time. The slope of the regression line represents the degree of temporal changes for individual genes.

(B) Plot of the slope distribution for all genes with an average expression level over cutoff (baseMean>100, calculated in DESeq2). We chose the genes with the highest and lowest slopes (the top and bottom 10%) for pathway enrichment analysis.

Disease Models & Mechanisms • Supplementary information

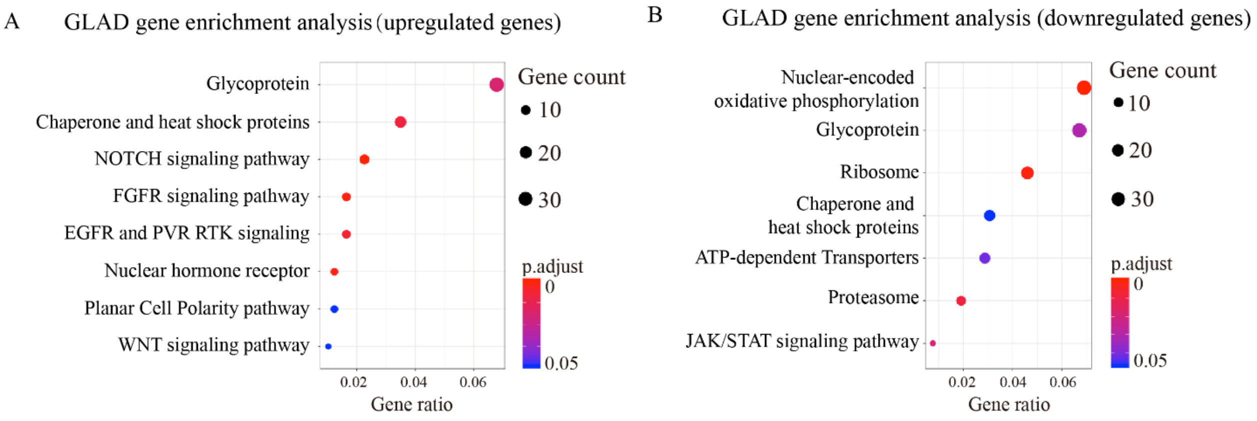

Figure S6. GLAD database pathway enrichment analysis for the top 10% upregulated genes (A) and the bottom 10% downregulated genes (B) over time with a cutoff of adjusted p-value< 0.05.

The pathway enrichment analysis is performed using the clusterProfiler package.

Disease Models & Mechanisms • Supplementary information

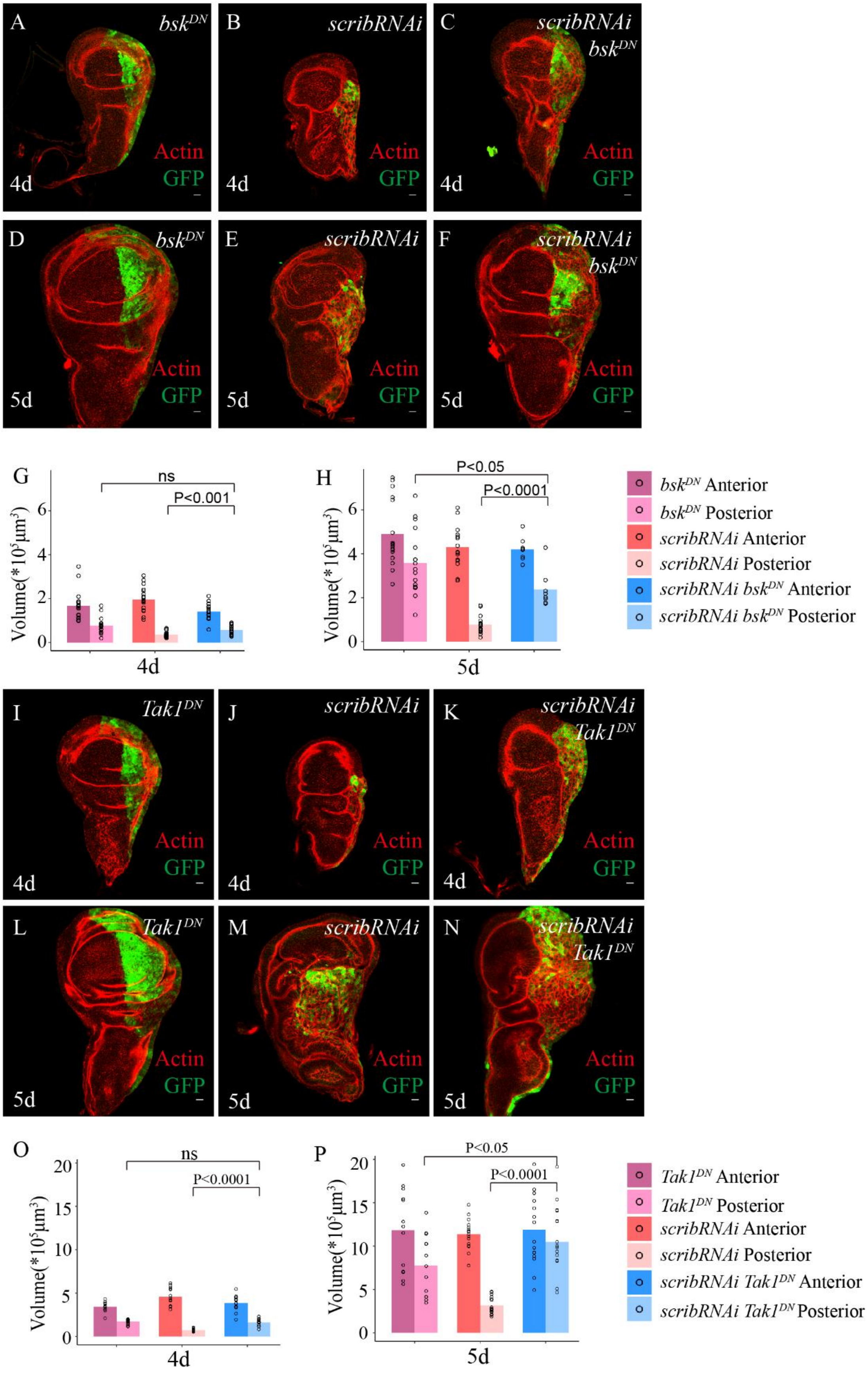

Disease Models & Mechanisms • Supplementary information

Figure S7. Overexpression of *bsk^{DN}* or *Tak1^{DN}* release the *scrib* RNAi cells from growth arrest.

(A-F) 4-day (A-C) and 5-day (D-F) AEL *bsk^{DN}* (A, D), *scrib RNAi* (B, E) and *scrib RNAi bsk^{DN}* (C, F) imaginal discs stained for actin(red) and GFP (green). Genotype for (A) and (D): *engrailed-Gal4 UAS-GFP/+; UAS- bsk^{DN}/+*. Genotype for (B) and (E): *engrailed-Gal4 UAS-GFP/UAS-scribRNAi*. Genotype of (C) and (F): *engrailed-Gal4 UAS-GFP/UAS-scribRNAi; UAS- bsk^{DN}/+*. Scale bar: 10µm.

(G-H) Quantification of volumes for 4-day (G) and 5-day (H) AEL *bsk^{DN}*, *scribRNAi* and *scribRNAi bsk^{DN}* imaginal discs. ***bsk^{DN}***, **4d** n = 17, anterior, 1.7±0.7x10^{5}µm^3, posterior, 8±4x10^{4}µm^3, **5d** n = 17, anterior, 5±2x10^{5}µm^3, posterior, 4±1x10^{5}µm^3. ***scribRNAi***, **4d** n = 20, anterior, 2.0±0.5x10^{5}µm^3, posterior, 4±1x10^{4}µm^3, **5d** n = 15, anterior, 4±1x10^{5}µm^3, posterior, 8±4x10^{4}µm^3. ***scribRNAi bsk^{DN}***, **4d** n = 14, anterior, 1.4±0.4x10^{5}µm^3, posterior, 6±2x10^{4}µm^3, **5d** n = 8, anterior, 4.2±0.5x10^{5}µm^3, posterior, 2.4±0.8x10^{5}µm^3. Statistical analysis was performed by unpaired t-test.

(I-N) 4-day (I-K) and 5-day (L-N) AEL *Tak1^{DN}* (I, L), *scrib RNAi* (J, M) and *scrib RNAi Tak1^{DN}* (K, N) imaginal discs stained for actin (red) and GFP (green). Genotype for (I) and (L): *engrailed-Gal4 UAS-GFP/UAS-Tak1^{DN}*. Genotype for (J) and (M): *engrailed-Gal4 UAS-GFP/+; UAS-scribRNAi/+*. Genotype of (K) and (N): *engrailed-Gal4 UAS-GFP/ UAS-Tak1^{DN}; UAS-scribRNAi/+*. Scale bar: 10µm.

(O-P) Quantification of volumes for 4-day (O) and 5-day (P) AEL *Tak1^{DN}*, *scribRNAi* and *scribRNAi Tak1^{DN}* imaginal discs. ***Tak1^{DN}***, **4d** n = 14, anterior, 3.4±0.5x10^{5}µm^3, posterior, 1.7±0.3x10^{5}µm^3, **5d** n = 13 anterior, 1.2±0.5x10^{6}µm^3, posterior, 8±4x10^{5}µm^3; ***scribRNAi***, **4d** n = 14, anterior, 5±1x10^{5}µm^3, posterior, 7±2x10^{4}µm^3, **5d** n = 16, anterior, 1.1±0.2x10^{6}µm^3, posterior, 3±1x10^{5}µm^3; ***scribRNAi Tak1^{DN}***, **4d** n = 13, anterior, 3.8±0.9x10^{5}µm^3, posterior, 1.6±0.4x10^{5}µm^3, **5d** n = 15, anterior, 1.2±0.4x10^{6}µm^3, posterior, 1.0±0.4x10^{6}µm^3. Statistical analysis was performed by unpaired t-test.

Disease Models & Mechanisms • Supplementary information

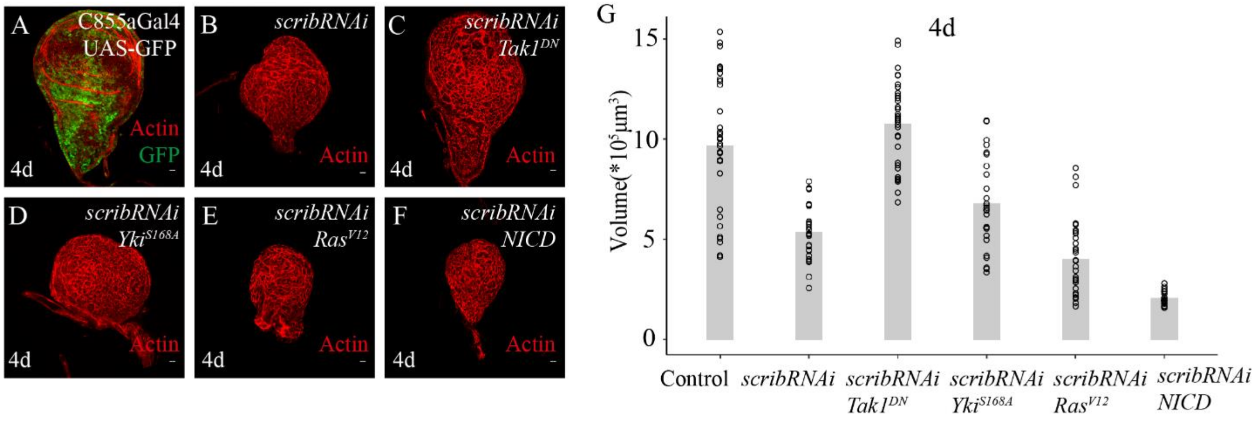

Figure S8. Overexpression of *Yki^{S168A}*, *Ras^{V12}* or *NICD* does not release the *scrib* RNAi cells from the early growth arrest stage.

(A-F) 4-day AEL control (A), *scribRNAi* (B), *scribRNAi Tak1^{DN}*(C), *scribRNAi Yki^{S168A}*(D), *scribRNAi Ras^{V12}*(E), and *scribRNAi NICD* (F) imaginal discs stained for actin (red). Genotypes are as follows: (A) *C855a-Gal4/UAS-GFP;* (B) *C855a-Gal4/UAS-scribRNAi;* (C) *UAS-Tak1^{DN}/+; C855a-Gal4/UAS-scribRNAi;* (D) *UAS-Yki^{S168A}/+; C855a-Gal4/UAS-scribRNAi;* (E) *UAS-Ras^{V12}/+; C855a-Gal4/UAS-scribRNAi;* (F) *UAS-NICD/+; C855a-Gal4/UAS-scribRNAi.* Scale bar: 10μm.

(G) Quantification of volumes for 4-day AEL control (n = 33, 1.0±0.3x10^{6}μm^{3}), *scribRNAi* (n = 30, 5±1x10^{5}μm^{3}), *scribRNAi Tak1^{DN}* (n = 35, 1.1±0.2x10^{6}μm^{3}), *scribRNAi Yki^{S168A}* (n = 26, 7±2 x10^{5}μm^{3}), *scribRNAi Ras^{V12}* (n = 30, 4±2x10^{5}μm^{3}), and *scribRNAi NICD* (n = 18, 2.1±0.4x10^{5}μm^{3}) imaginal discs.

Disease Models & Mechanisms • Supplementary information

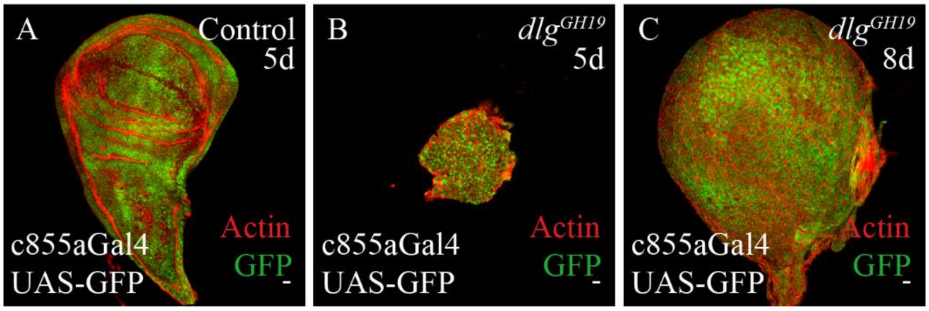

Figure S9. The expression pattern of c855a-Gal4 driver in the wild-type and the *dlg* mutant imaginal discs.

(A-C) 5-day AEL control (A), 5-day AEL (B) and 8-day AEL *dlg* mutant (C) imaginal discs stained for actin (red). Genotype for (A): UAS-GFP/+; c855a-Gal4/+. Genotype for (B) and (C): *dlg*^{GH19}/Y; UAS-GFP/+; c855a-Gal4/+. Scale bar: 10µm.

Disease Models & Mechanisms • Supplementary information

A

Gene	4d	5d	8d	14d
Fim	0.628	0.590	0.485	0.282
cindr	0.615	0.615	0.432	0.289
zip	0.580	0.579	0.490	0.406
Mlc-c	0.544	0.567	0.472	0.360
Vinc	0.540	0.537	0.397	0.282
Arpc3A	0.540	0.523	0.428	0.325
Tm1	0.534	0.559	0.484	0.389
sqh	0.533	0.572	0.497	0.442
Arpc1	0.531	0.558	0.436	0.334
Act5C	0.525	0.542	0.474	0.327

B

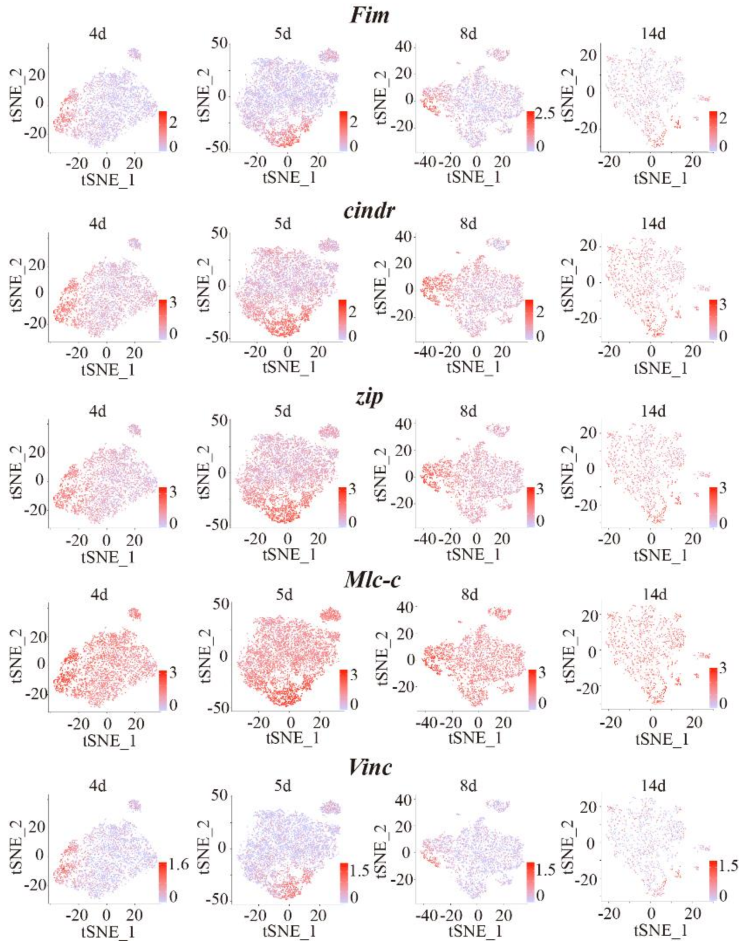

Figure S10. A number of cytoskeletal genes shows high expression correlation with *Mmp1* from the *scrib* mutant single cell transcriptomic analysis.

(A) List of the top ten cytoskeletal genes the expression of which shows high correlation with *Mmp1* in the *scrib* mutant tumors. The Pearson correlation coefficient is calculated using normalized gene expression levels in 4-day, 5-day, 8-day and 14-day AEL $scrib^1$ mutant single cells.

(B) t-SNE projection of 4-day, 5-day, 8-day and 14-day AEL $scrib^1$ mutant cells where individual cell is colored by the normalized expression level of *Fim*, *cindr*, *zip*, *Mlc-c*, and *Vinc*. Note that their expression patterns are similar to the *Mmp1* expression pattern in the t-SNE plot in Figure 3I. The gene expression level normalization is performed using the default LogNormalize method in Seurat.

Disease Models & Mechanisms • Supplementary information

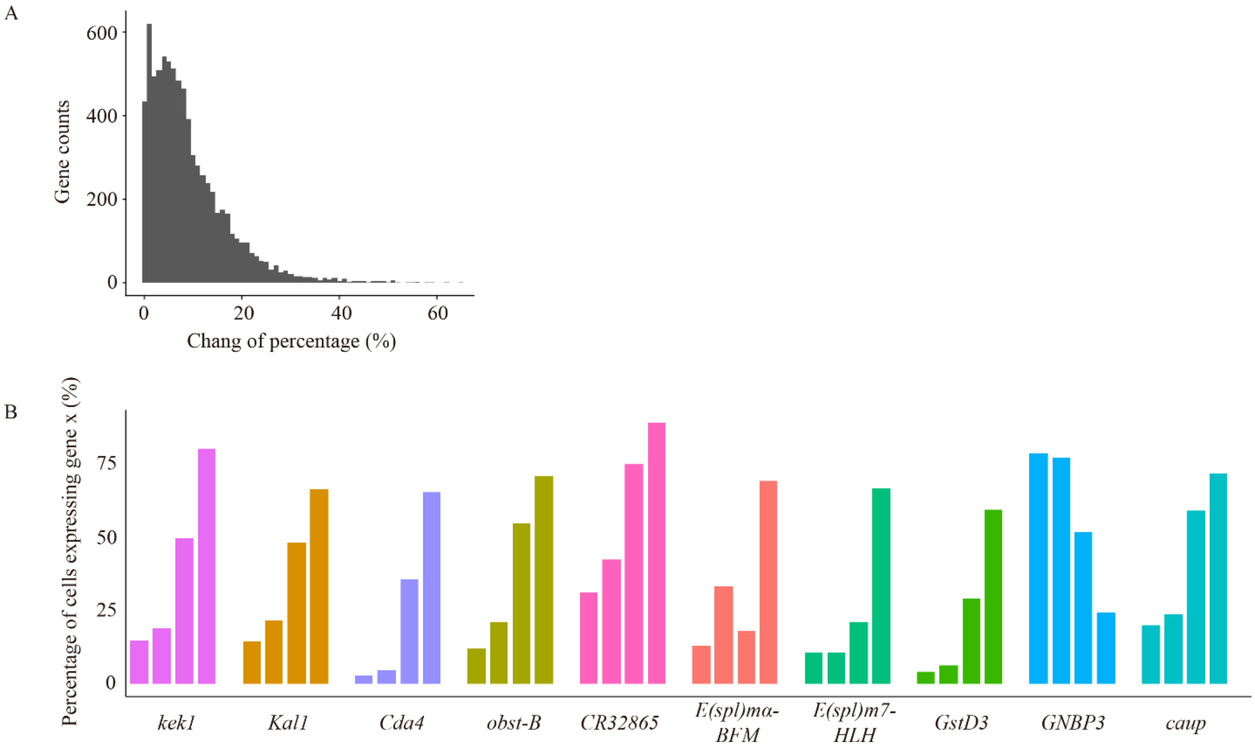

Figure S11. The percentage of *kek1*+ cells increases significantly in the *scrib* mutant tumors over time.

(A) Plot of distribution of absolute percentage change between 4-day and 14-day AEL *scrib* mutant tumors for all expressed genes. For any given gene detected in both 4-day and 14-day AEL *scrib* mutant tumors, we calculated the percentages of cells expressing that specific gene in 4-day and 14-day AEL *scrib* mutant tumors respectively and obtained the absolute value of the difference between two percentages as the absolute percentage change for that specific gene.

(B) For the top 10 genes with significant changes in the percentage of cells expressing the specific gene over time, the percentages of cells expressing that gene (normalized expression value >0) in the 4-day, 5-day, 8-day and 14-day *scrib* mutant tumors over time are plotted.

Disease Models & Mechanisms • Supplementary information

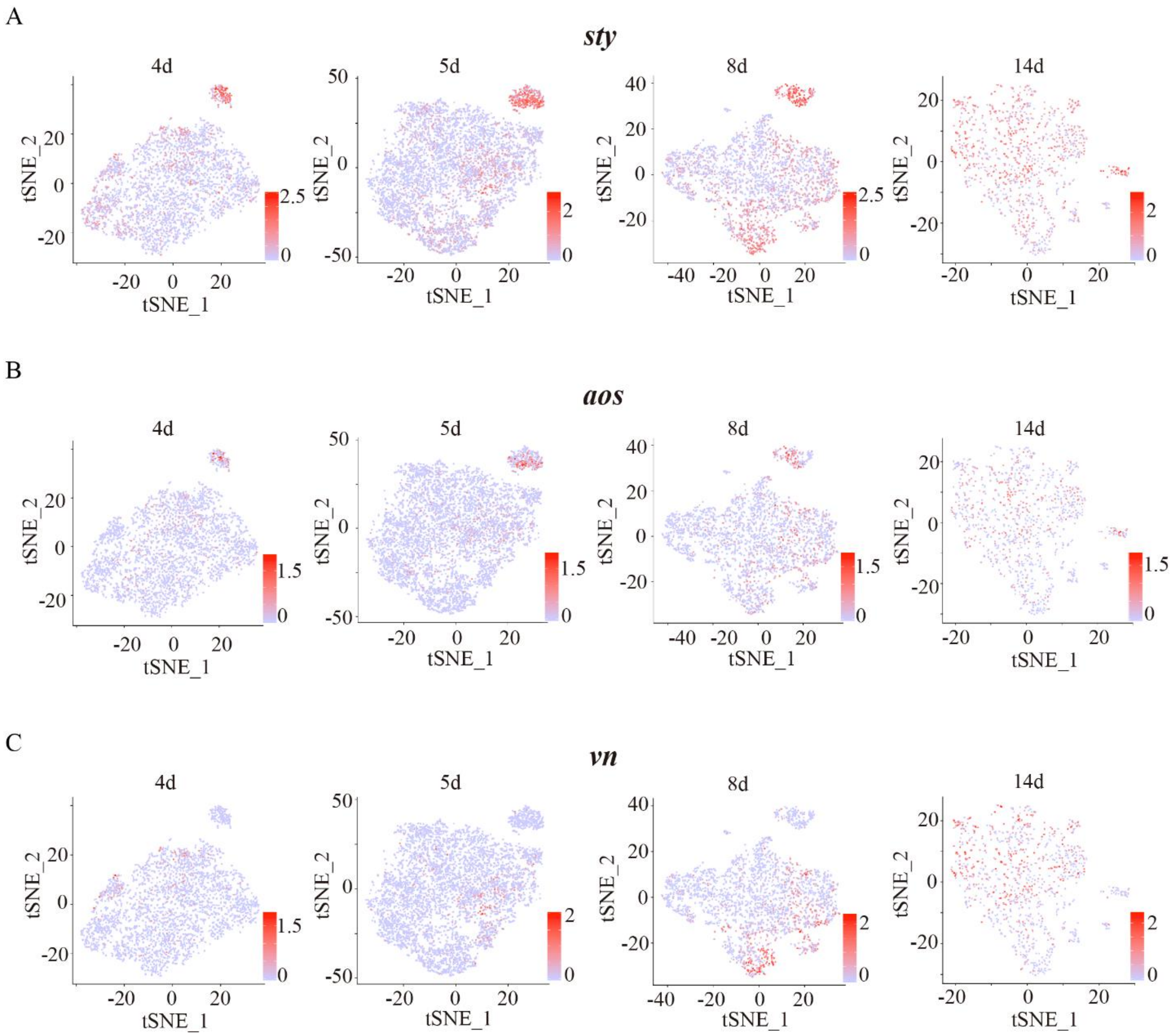

Figure S12. t-SNE projection of 4-day, 5-day, 8-day and 14-day AEL *scrib*^{1} mutant cells where individual cell is colored by the normalized expression level of *sty, aos and vn*. The gene expression level normalization is performed using the default LogNormalize method in Seurat.

Disease Models & Mechanisms • Supplementary information

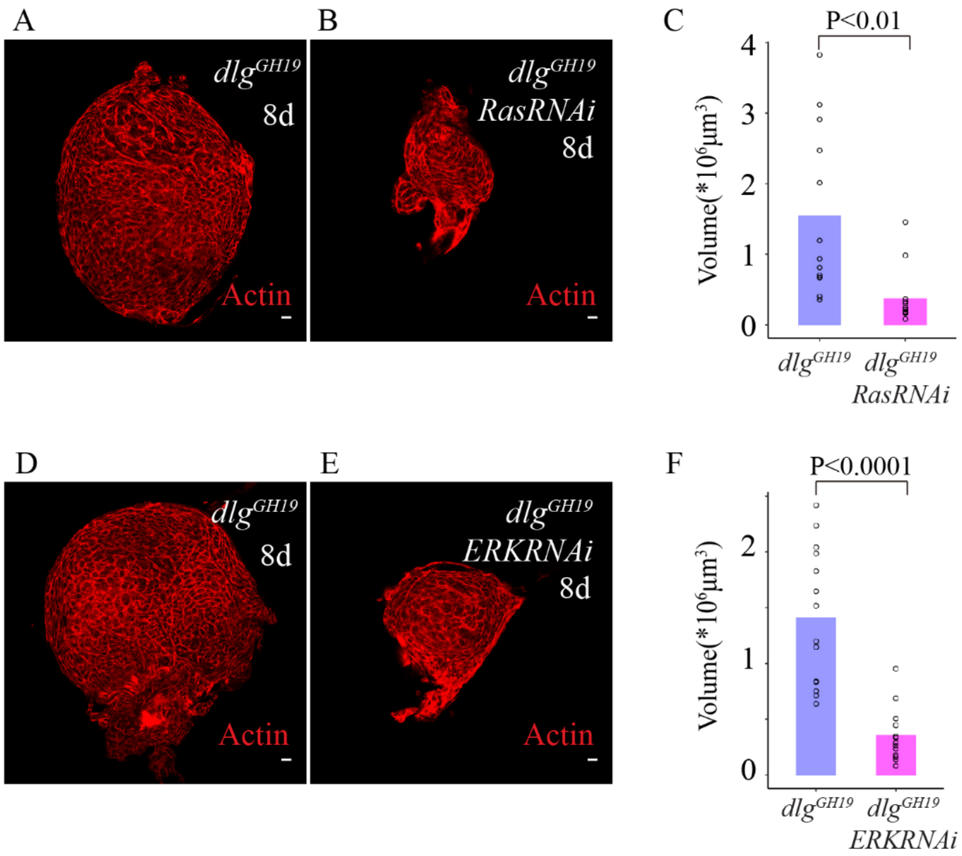

Figure S13. Blocking ERK signaling activity leads to a reduction of the *dlg* mutant tumor size at later stages.

(A-C) A 8-day AEL *dlg*^{GH19} (A) and *dlg*^{GH19} *Ras RNAi* (B) imaginal disc stained for actin (red). Quantifications of tumor volumes are plotted in (C). Genotype for (A): *dlg*^{GH19}/Y; *c855a-Gal4/+*, n=13, 2±1x10^{6}µm^{3}. Genotype for (B): *dlg*^{GH19}/Y; *c855a-Gal4/ UAS-Ras RNAi*, n=13, 4±4x10^{5}µm^{3}. Scale bar: 10µm. Statistical analysis was performed by unpaired t-test.

(D-F) A 8-day AEL *dlg*^{GH19} (D) and *dlg*^{GH19} *ERK RNAi* (E) imaginal disc stained for actin (red). Quantifications of tumor volumes are plotted in (F). Genotype for (D): *dlg*^{GH19}/Y; *c855a-Gal4/+*, n=14, 1.4±0.6x10^{6}µm^{3}. Genotype for (E): *dlg*^{GH19}/Y; *c855a-Gal4/ UAS-ERK RNAi*, n=13, 4±2x10^{5}µm^{3}. Scale bar: 10µm. Statistical analysis was performed by unpaired t-test.

Disease Models & Mechanisms • Supplementary information

A

Gene	4d	5d	8d	14d
Sulf1	0.552	0.567	0.391	0.235
drk	0.517	0.527	0.330	0.205
Fas2	0.446	0.400	0.270	0.303
rho-4	0.373	0.347	0.279	0.201
Vps4	0.360	0.380	0.301	0.182

B

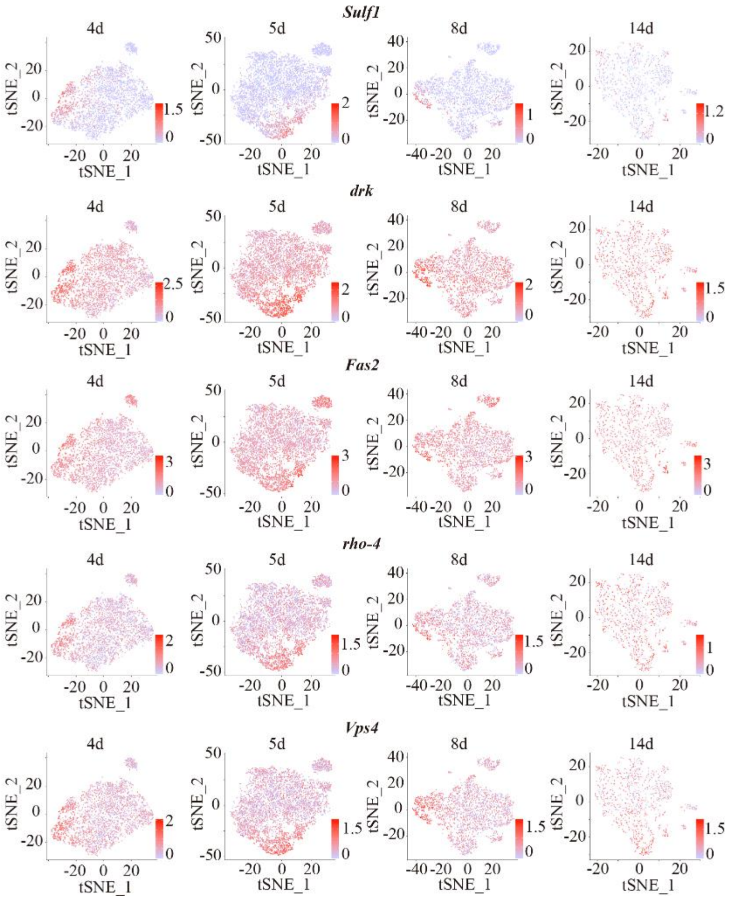

Figure S14. Several ERK pathway genes shows high expression correlation with *Mmp1* from the *scrib* mutant single cell transcriptomic analysis.

(A) List of 5 ERK pathway genes the expression of which shows high correlation with *Mmp1* in the *scrib* mutant tumors. The Pearson correlation coefficient is calculated using normalized gene expression levels in 4-day, 5-day, 8-day and 14-day AEL *scrib*^{1} mutant single cells.

(C) t-SNE projection of 4-day, 5-day, 8-day and 14-day AEL *scrib*^{1} mutant cells where individual cell is colored by the normalized expression level of *Sulf1, drk, Fas2, rho-4* and *Vps4*. Note that their expression patterns are similar to the *Mmp1* expression pattern in the t-SNE plot in Figure 3I. The gene expression level normalization is performed using the default LogNormalize method in Seurat.

Disease Models & Mechanisms • Supplementary information

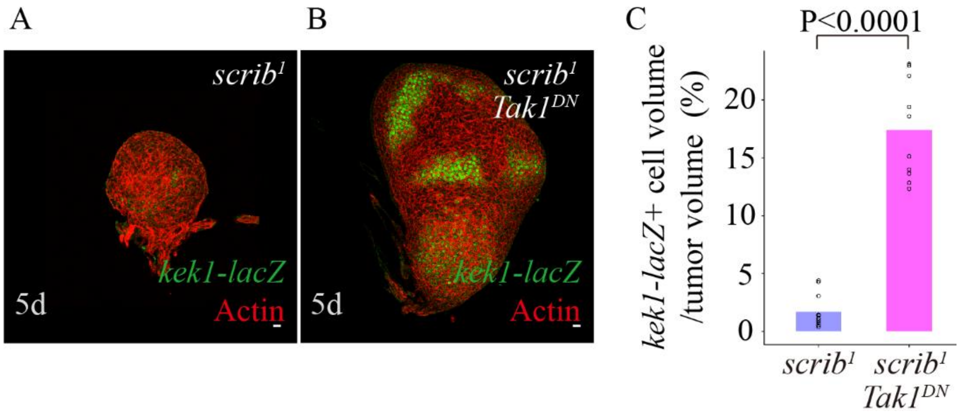

Figure S15. High JNK signaling activity possibly represses ERK activity in the early *scrib* mutants.

(A-C) A 5-day AEL *scrib*^{1} (A) and *scrib*^{1} *Tak1*^{DN} (B) imaginal disc stained for actin (red). Quantifications of *kek1-lacZ+* cell volume/tumor volume are plotted in (C). Genotype for (A): *kekl-lacZ/+; scrib*^{1} *c855a-Gal4/ scrib*^{1}. Genotype for (B): *kekl-lacZ/UAS-Tak1*^{DN}*; scrib*^{1} *c855a-Gal4/ scrib*^{1}. Scale bar: 10µm. Statistical analysis was performed by unpaired t-test.

Disease Models & Mechanisms • Supplementary information

Table S1. List of Pearson correlation coefficients for all expressed genes with *Mmp1* in 4-day, 5-day, 8-day and 14-day AEL *scrib*^{1} mutant single cells.

Click here to Download Table S1

Disease Models & Mechanisms • Supplementary information

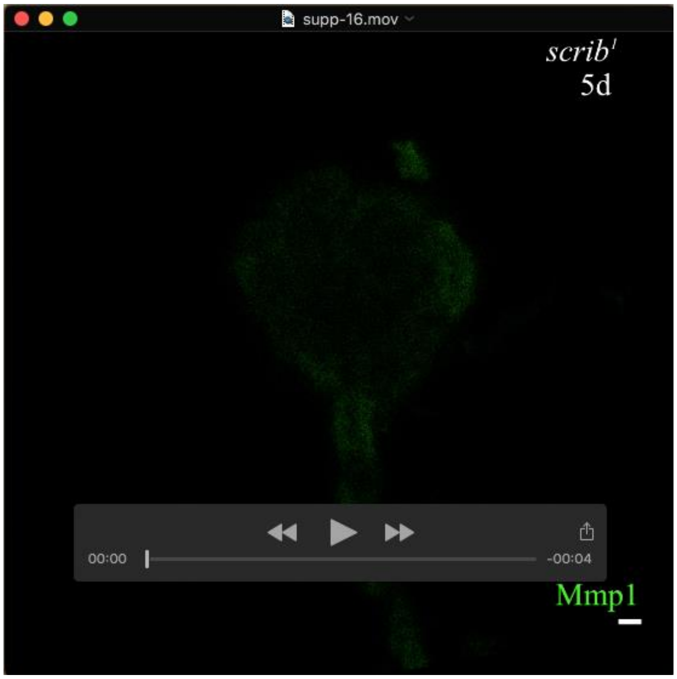

Movie 1. Z-stack images of a 5-day *scrib*[1] mutant tumor stained for Mmp1 (Green). The z-stacks was taken at 1µm interval. Scale bar: 10µm.

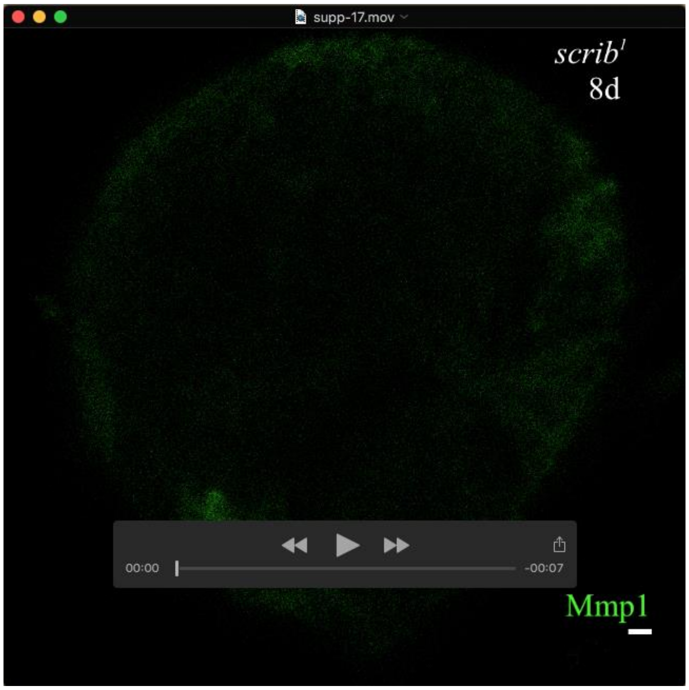

Movie 2. Z-stack images of an 8-day *scrib*^{1} mutant tumor stained for Mmp1 (Green). The z-stacks was taken at 1µm interval. Scale bar: 10µm.

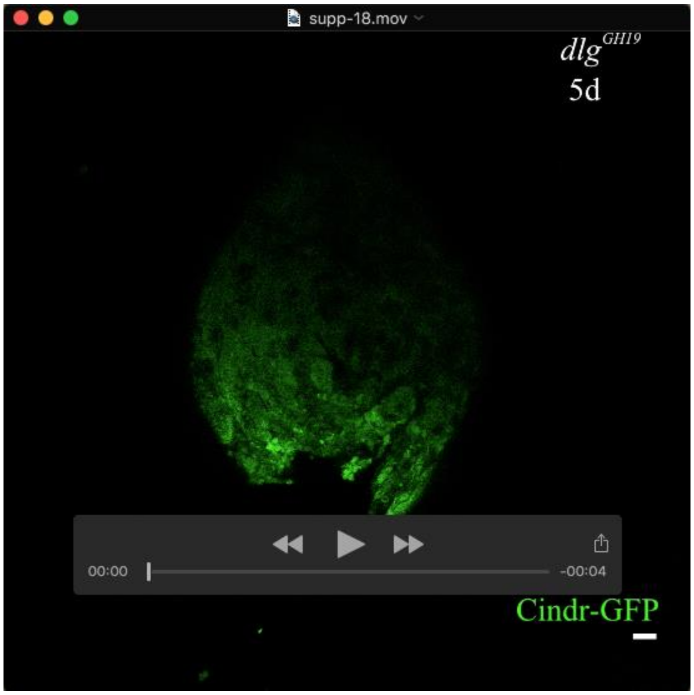

Movie 3. Z-stack images of a 5-day *dlg*^{GH19}*; Cindr-GFP* mutant tumor. The z-stacks was taken at 1µm interval. Scale bar: 10µm.

Disease Models & Mechanisms • Supplementary information

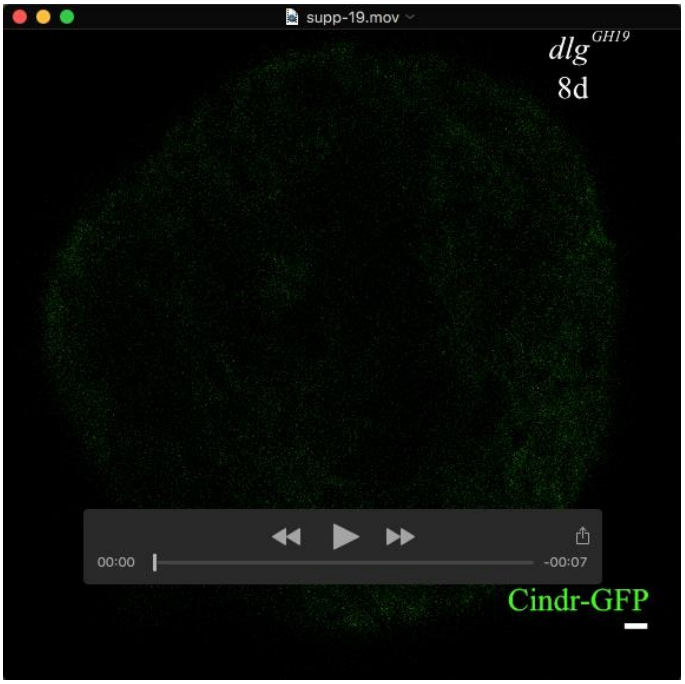

Movie 4. Z-stack images of an 8-day *dlg*^{GH19}*; Cindr-GFP* mutant tumor. The z-stacks was taken at 1µm interval. Scale bar: 10µm.

Disease Models & Mechanisms • Supplementary information

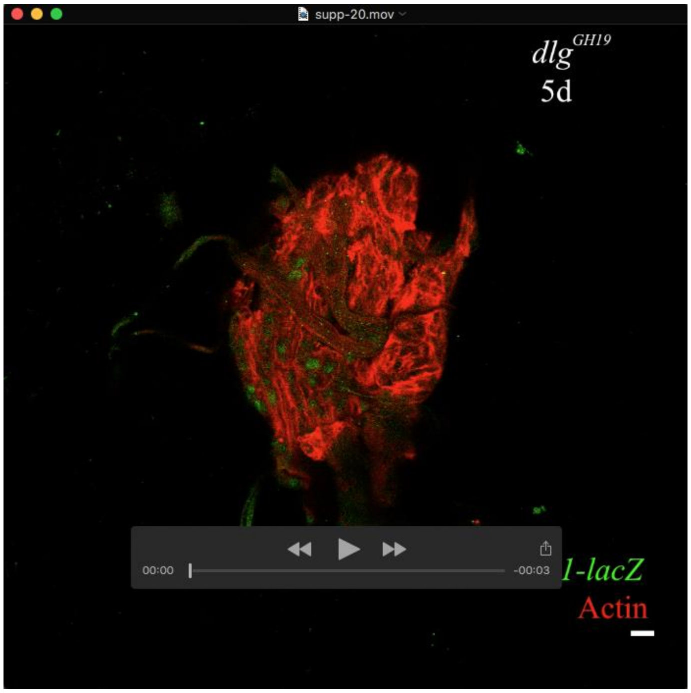

Movie 5. Z-stack images of a 5-day *dlg^{GH19}; kek1-lacZ; c885aGal4/+* mutant tumor stained for β-gal (green) and actin (red). The z-stacks was taken at 1μm interval. Scale bar: 10μm.

Disease Models & Mechanisms • Supplementary information

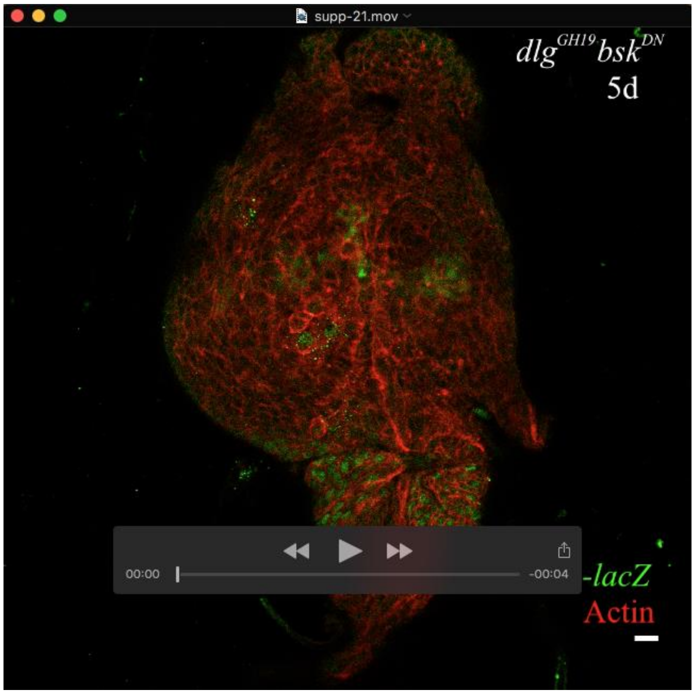

Movie 6. Z-stack images of a 5-day *dlg^{GH19}; kek1-lacZ; c885aGal4/UAS-bsk^{DN}* mutant tumor stained for β-gal (green) and actin (red). The z-stacks was taken at 1μm interval. Scale bar: 10μm.
